# Nuclear import of PTPN18 inhibits breast cancer metastasis mediated by MVP and importin β2

**DOI:** 10.1038/s41419-022-05167-z

**Published:** 2022-08-18

**Authors:** Tao Wang, Xinlei Ba, Xiaonan Zhang, Na Zhang, Guowen Wang, Bin Bai, Tong Li, Jiahui Zhao, Yanjiao Zhao, Yang Yu, Bing Wang

**Affiliations:** 1 College of Life and Health Sciences, Northeastern University, Shenyang, Liaoning P. R. China; 2 Department of Pathophysiology, Bengbu Medical College, Bengbu, Anhui P. R. China; 3 Department of Thoracic surgery, The First Affiliated Hospital of Bengbu Medical College, Bengbu, Anhui P. R. China

**Keywords:** Breast cancer, Epithelial-mesenchymal transition

## Abstract

Distant metastasis is the primary cause of breast cancer-associated death. The existing information, such as the precise molecular mechanisms and effective therapeutic strategies targeting metastasis, is insufficient to combat breast cancer. This study demonstrates that the protein tyrosine phosphatase PTPN18 is downregulated in metastatic breast cancer tissues and is associated with better metastasis-free survival. Ectopic expression of PTPN18 inhibits breast cancer cell metastasis. PTPN18 is translocated from the cytoplasm to the nucleus by MVP and importin β2 in breast cancer. Then, nuclear PTPN18 dephosphorylates ETS1 and promotes its degradation. Moreover, nuclear PTPN18 but not cytoplasmic PTPN18 suppresses transforming growth factor-β signaling and epithelial-to-mesenchymal transition by targeting ETS1. Our data highlight PTPN18 as a suppressor of breast cancer metastasis and provide an effective antimetastatic therapeutic strategy.

## Introduction

Breast cancer is the most common carcinoma in females worldwide [1], and metastasis is the leading cause of death in breast cancer patients [2]. During metastasis, cancer cells undergo advanced genetic/epigenetic and phenotypic modifications, which cause their dispersion from primary tumors, subsequent intravasation and migration, and ultimate colonization in distant organs [3, 4]. At the molecular level, the epithelial-to-mesenchymal transition (EMT) process generates dynamic cellular heterogeneity during metastasis [5], whereby tumor cells progressively lose their polarity and adhesion capacity but become more migratory and invasive [6]. One of the most challenging aspects of targeted breast cancer treatment is coping with the wide range of spatial and temporal heterogeneity in primary tumors [7, 8]. Thus, the molecular mechanism underlying this spatiotemporal regulation of breast cancer metastasis merits in-depth investigation.

In eukaryotic cells, many biological processes, including cancer metastasis, are regulated by nuclear transport [9–11]. Nuclear import of proteins with a mass of >40 kDa is dependent on the presence of nuclear localization signals (NLSs) [12]. The best-characterized import pathway is mediated by canonical NLSs, which are short, lysine/arginine-rich motifs that interact with importin α and heterodimerize with importin β [13, 14]. Other proteins imported into the nucleus do not rely on a canonical pathway but bind directly to importin β [15]. Interestingly, nuclear localization has also been identified to involve MVP, the major component of vaults, which are the largest ribonucleoprotein particles and feature a hollow barrel-like structure [16]. MVP was hypothesized to be a general carrier molecule for nuclear-cytoplasmic transport [17] and has recently been reported to promote nuclear entry of PTEN and GLI1 through interaction with NLSs [18, 19]. However, the mechanism of MVP-mediated nuclear import is still unclear.

PTPN18 (also called PTP-HSCF or BDP1), a member of the protein tyrosine phosphatase superfamily [20], has been reported to be a tumor suppressor in breast cancer by dephosphorylating HER2 and suppressing cell proliferation and invasion [21, 22]. In addition to the well-established role of PTPN18 in breast cancer, our previous study found that PTPN18 and CSK cooperate to inhibit the events triggered by SRC kinases [23]. While these studies established PTPN18 as a suppressive regulator of breast cancer progression, the molecular mechanism of PTPN18 in breast cancer metastasis remains to be clarified. Here, we identified a unique function of PTPN18 in inhibiting EMT, TGFβ signaling, and cell motility. PTPN18 is downregulated in metastatic breast cancer tissue and predicts lung metastasis-free survival in patients with breast cancer. PTPN18 localizes in both the nucleus and cytoplasm in breast cancer cells. Nuclear import of PTPN18 is mediated by MVP and importin β2 via interactions with the functional NLSs of PTPN18. PTPN18 dephosphorylates and promotes the degradation of ETS1 in the nucleus. Our data delineate the nuclear translocation of PTPN18 and identify nuclear PTPN18 as a novel antimetastatic therapeutic target.

## Results

### PTPN18 interacts with MVP in vitro and in vivo

**Fig. 1:**
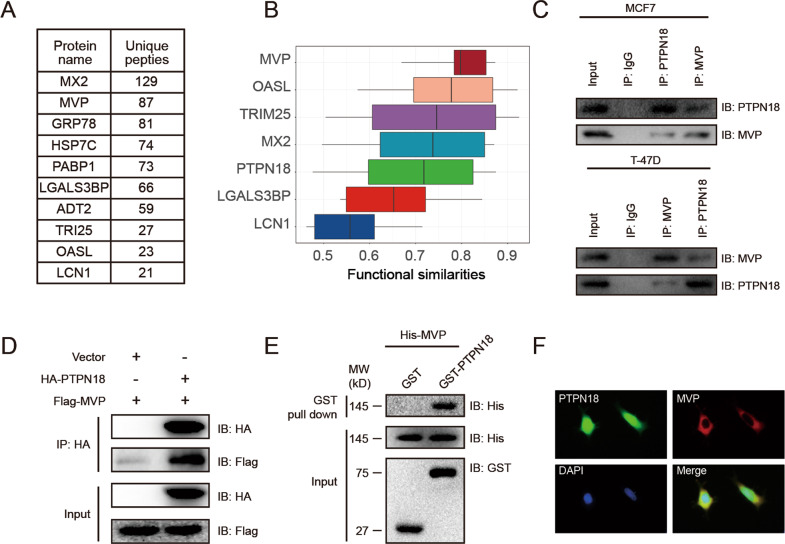
PTPN18 interacts with MVP in vitro and in vivo. **A** Proteins identified in the immunopurified PTPN18 complexes by mass spectrometry. **B** Summary of functional similarities of the PTPN18 interactome in MCF7 cells. **C** The physical association between endogenous PTPN18 and MVP was validated using immunoprecipitation (IP) in MCF7 and T-47D cells. **D** Co-IP showed the interaction between ectopic PTPN18 and MVP in 293 T cells. **E** GST pull-down assay with GST-tagged PTPN18 and His-tagged MVP. **F** Representative immunofluorescence images showing the colocalization of PTPN18 (green) and MVP (red) in MCF7 cells.

PTPN18 has been described to be expressed in breast cancer cells and implicated in diverse cellular functions, such as regulation of HER2 signaling, dynamic rearrangements of the cytoskeleton, and activation of the MYC-CDK4 axis [22, 24–26]. We sought to identify the primary interaction partners of PTPN18 in breast cancer cells and explore its novel molecular functions. Utilizing a proteomic approach consisting of immunoprecipitation of PTPN18 followed by LC-MS/MS analysis, we identified several proteins that interact with PTPN18, and the ten proteins with the highest unique peptide numbers determined by MS are listed in Fig. 1A. To identify essential proteins in the PTPN18 interactome in MCF7 cells, we ranked proteins by their average functional similarity [27]. Functional similarity, defined as the geometric mean of the semantic similarities of paired proteins in the molecular function and cellular component aspect of Gene Ontology, is designed to evaluate the strength of the relationship between each protein and its interaction partners by considering the function and localization of the proteins [28]. MVP presented the highest average functional similarity, indicating the potential molecular functions of PTPN18 mediated by MVP (Fig. 1B).

To demonstrate the physical association between PTPN18 and MVP, we applied an immunoprecipitation assay with endogenous PTPN18 and MVP. We found that endogenous PTPN18 or MVP coprecipitated with endogenous MVP or PTPN18, respectively, in lysates of MCF7 and T-47D cells (Fig. 1C). Similar results were observed for the interaction between ectopic PTPN18 and MVP using coimmunoprecipitation in 293 T cells (Fig. 1D). We also performed a GST pull-down assay to confirm their direct interaction (Fig. 1E). Finally, the colocalization of PTPN18 (green) and MVP (red) was observed in situ by fluorescence microscopy (Fig. 1F). These results indicated that PTPN18 interacts with MVP in breast cancer cells, and that the function of their interaction is worth in-depth investigation.

### Two functional NLSs in the catalytic domain of PTPN18 are required for its nuclear localization

**Fig. 2:**
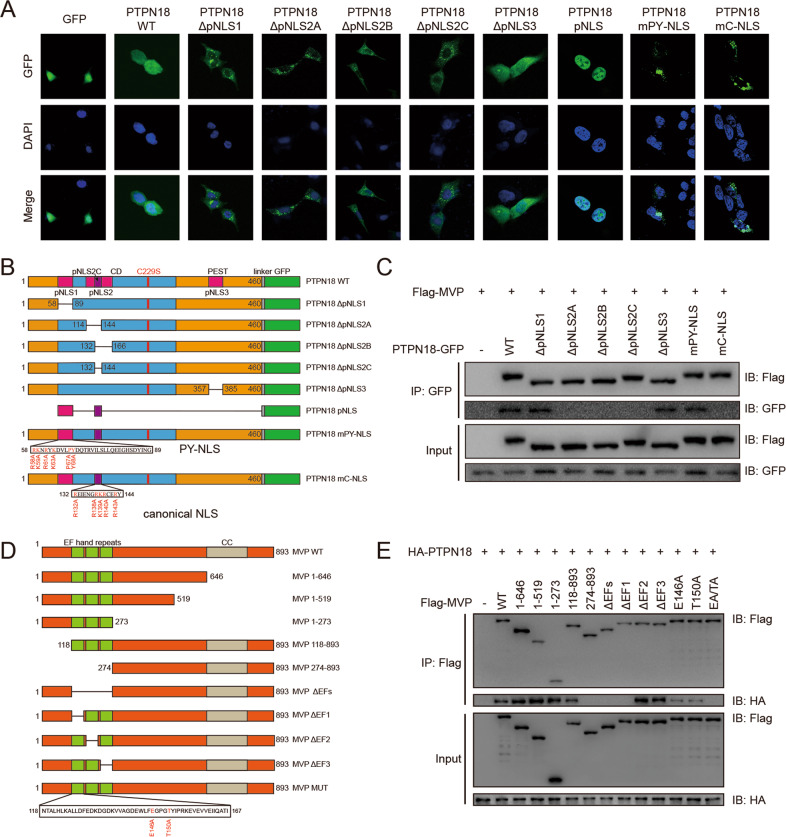
Investigation of crucial regions and residues mediating the PTPN18-MVP interaction. **A** Subcellular localization of different PTPN18-GFP truncations in MCF7 cells. **B** Schematic representation of different PTPN18 truncations based on their putative NLS domains. **C** Co-IP of Flag-MVP with different truncated forms of PTPN18-GFP in 293 T cells. **D** Schematic representation of different MVP truncations based on their functional domains. **E** Co-IP of HA-PTPN18 with different truncated forms of Flag-MVP in 293 T cells.

As MVP can function as a nuclear import carrier by binding to NLSs of target cargo proteins [18] and PTPN18 is localized subcellularly in the nucleus and cytoplasm (Fig. 1F), we hypothesized that MVP might interact with the NLS of PTPN18 and be involved in PTPN18 nuclear import. To substantiate this hypothesis, we predicted the NLSs of PTPN18 using cNLS Mapper [29]. Four putative NLSs, including two overlapping regions, were identified (Fig. S1A). Then, GFP-tagged PTPN18 truncations were generated to explore the interaction domain and examine whether the putative NLSs are required for the PTPN18 nuclear localization. The immunofluorescence results showed that PTPN18 WT and the ΔpNLS3 mutant were distributed in the nucleus and cytoplasm, whereas the ΔpNLS1 and ΔpNLS2-related PTPN18 truncations were accumulated exclusively in the cytoplasm (Fig. 2A). These observations indicated that the regions of PTPN18 encompassing residues 58–89 and 132–144 are responsible for its nuclear localization, and two functional NLSs were thus identified in the catalytic domain of PTPN18.

To further test whether the two identified NLSs (pNLS1 and pNLS2C) are sufficient to mediate nuclear localization, we constructed additional fragments: PTPN18 pNLS (a fusion protein containing pNLS1, pNLS2C, and a GFP tag), mPY-NLS (in which all arginines and lysines in the region from R58 to K63, as well as P67 and Y68 were mutated to Ala) and mC-NLS (in which all arginines and lysines in the region from R132 to Y144 were mutated to Ala) (Fig. 2B) [14, 30]. PTPN18 pNLS was imported into the nucleus, whereas PTPN18 mPY-NLS and mC-NLS were only localized in the cytoplasm and the perinuclear region (Fig. 2A). These data suggested that PTPN18 contains one C-NLS and one PY-NLS required for PTPN18 nuclear import, and these sequences were found to be conserved across species (Fig. S1B). The corresponding two mutants, PTPN18 mPY-NLS and mC-NLS, were defined as cytoplasmic PTPN18 for further research.

### The canonical NLS of PTPN18 mediates the interaction with the E146 and T150 residues of MVP

We subsequently identified which NLS of PTPN18 was responsible for interacting with MVP. In vivo binding assays showed that PTPN18 ΔpNLS2A-C and mC-NLS could not bind MVP while PTPN18 ΔpNLS1, ΔpNLS3, and mPY-NLS bound MVP as effectively as PTPN18 WT (Fig. 2C), indicating the indispensable role of C-NLS in the PTPN18-MVP interaction.

To explore the residues of MVP essential for the MVP-PTPN18 interaction, we constructed MVP truncations according to their putative structural and functional domains [31, 32] (Fig. 2D). In vivo binding studies confirmed that PTPN18 bound stoichiometrically to MVP fragments that contained the first EF-hand domain (Fig. 2E). We further constructed a series of MVP point mutations based on the difference among the three EF-hand repeats [33] and tested the interactions of these mutants with PTPN18. We identified two residues (E146 and T150) indispensable for the MVP-PTPN18 interaction (Fig. 2E). Together, these results indicate that PTPN18 associates with MVP through the C-NLS of PTPN18 and the E146/T150 sites of MVP and that the nuclear localization of PTPN18 requires both the C-NLS and PY-NLS.

### MVP and importin β2 cooperatively promote PTPN18 nuclear import

**Fig. 3:**
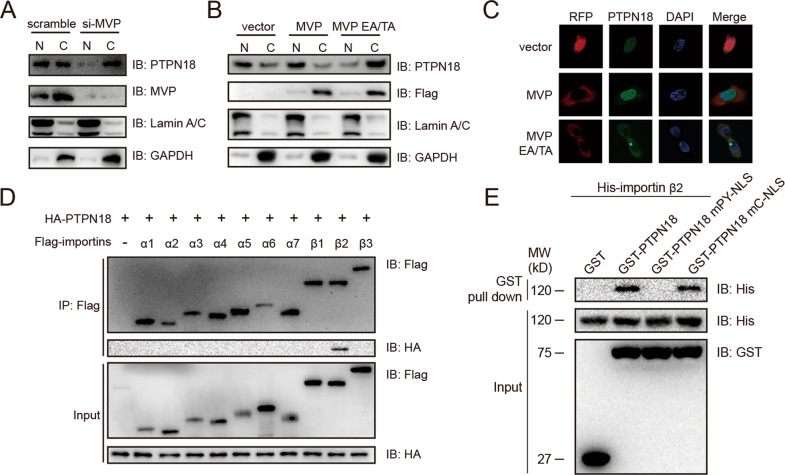
MVP and importin β2 cooperate to facilitate PTPN18 nuclear localization. **A** Immunoblot of the PTPN18 nucleocytoplasmic distribution after transient knockdown of MVP. N nucleus; C cytoplasm. **B** Immunoblot of the PTPN18 nucleocytoplasmic distribution after ectopic expression of MVP and its mutants. N nucleus; C cytoplasm. **C** Immunofluorescence analysis of the PTPN18 nucleocytoplasmic distribution in MCF7 cells after ectopic expression of MVP and its mutants. **D** Interactions between PTPN18 and importin family were examined by Co-IP. **E** GST pull-down assay of GST-tagged PTPN18 and its NLS mutants with His-tagged importin β2.

Since MVP interacts with the C-NLS of PTPN18, which is essential for its nuclear localization, we then evaluated the effect of MVP and its mutants on the nucleocytoplasmic shuttling of PTPN18. We found that the PTPN18 abundance was reduced in the nucleus but increased in the cytoplasm after MVP knockdown (Fig. 3A). Conversely, ectopic expression of MVP enhanced the nuclear translocation of PTPN18. However, expression of the MVP EA/TA mutant, which lost the ability to bind to PTPN18, significantly inhibited PTPN18 translocation from the cytoplasm to the nucleus (Fig. 3B, C).

MVP exclusively interacts with the C-NLS but not the PY-NLS, suggesting that multiple pathways may be involved in this translocation. Importins are responsible for transporting NLS-containing macromolecular cargo from the cytoplasm to the nucleus [34]. To determine whether the nuclear localization of PTPN18 is associated with the importin α/β system, RFP-Bimax2 and RFP-M9M, specific inhibitors of importin α and importin β2 (recognizing the PY-NLS), respectively [35, 36], were used to monitor the subcellular localization of PTPN18. Subcellular fractionation and fluorescence microscopy analyses showed that the M9M peptide markedly prevented PTPN18 nuclear import (Fig. S1C, D), strengthening the hypothesis that importin β2 is important in PTPN18 nuclear translocation. To further evaluate the interaction between PTPN18 and importin α/β, we detected the expression of importin genes in breast cancer (Fig. S1E) and used immunoprecipitation to test their binding to PTPN18. PTPN18 precipitated with importin β2 but not importin α1–7, β1, or β3 (Fig. 3D). We also identified the direct interaction between PTPN18 and importin β2 using purified proteins and found that PTPN18 WT and mC-NLS but not mPY-NLS interacted with importin β2 in vitro (Fig. 3E). Consistent with the binding assay results, knockdown of importin β2 effectively disrupted the translocation of PTPN18 from the cytoplasm to the nucleus (Fig. S1F), indicating the indispensable role of importin β2 in the nuclear translocation of PTPN18. These results demonstrated that MVP and importin β2 cooperatively deliver PTPN18 into the nucleus.

### PTPN18 is associated with breast cancer metastasis

Given that metastasis is the main contributor to breast cancer mortality and considering the suppressive role of PTPN18 in breast cancer, we explored the exact function of PTPN18 in breast cancer metastasis. PTPN18 was inversely associated with distant metastasis, lung metastasis, and poor prognosis in breast cancer (Fig. S2A–C). Analysis of integrated datasets demonstrated that the gene expression of PTPN18 was significantly downregulated in metastases compared to normal tissues and primary tumors (Fig. S3A), which prompted further investigation of its expression level in human tissues. Interestingly, PTPN18 was not significantly different in primary breast cancer tissues with and without metastasis (Fig. S3B). However, PTPN18 was dramatically downregulated in lymph node metastases compared to the paired primary tumors at both mRNA and protein levels (Fig. S3C–E). In keeping with the clinical data and human tissue analyses, PTPN18 expression was negatively associated with metastatic capacity in multiple breast cancer cell lines (Fig. S3F–H) [37]. These results suggested a strong association between PTPN18 and human breast cancer metastasis.

### Nuclear PTPN18 dephosphorylates ETS1 and promotes its degradation

To predict the substrates of PTPN18 in the nucleus, we determined the correlations between PTPN18 and metastasis-related genes in the TCGA-BRCA dataset by Spearman correlation analysis (Fig. S4A–C). The transcription factor targeting these significant metastasis-related genes were defined as the potential targets of PTPN18. We were particularly interested in ETS1, a member of the ETS transcription factor family. ETS1 contributes to invasion by upregulating MMPs in MCF7 cells [38]. Moreover, ETS1 can increase the expression of ZEB1 by interacting with its promoter in cooperation with Twist [39] and induce EMT in cooperation with Slug [40, 41]. However, the functions of ETS1 regulated by PTPN18 are still unknown.

**Fig. 4:**
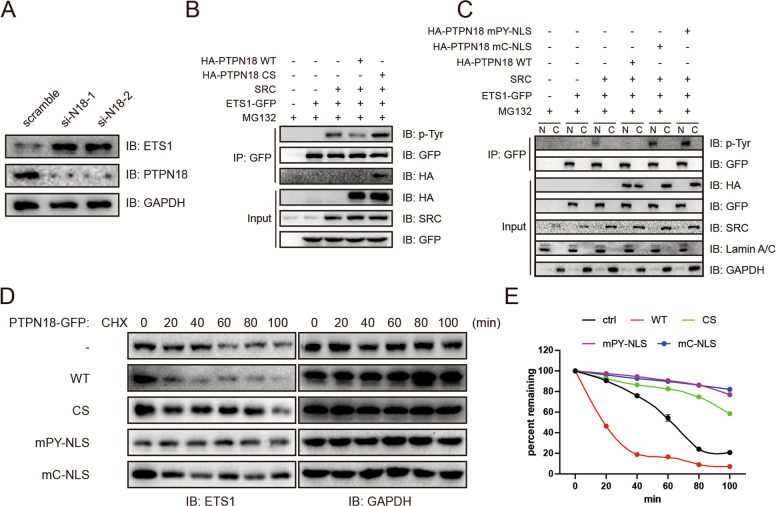
PTPN18 promotes ETS1 ubiquitination and degradation through regulation of its phosphorylation. **A** Immunoblot analysis of ETS1 expression after PTPN18 knockdown. **B** Immunoblot analysis of ETS1 tyrosine phosphorylation after ectopic expression of PTPN18 WT and mutants. Cells were treated with 10 mM MG132 6 h before harvesting. **C** Immunoblot analysis of ETS1 tyrosine phosphorylation after ectopic expression of PTPN18 WT or cytoplasmic PTPN18. Cells were treated with 10 mM MG132 6 h before harvesting. **D** Immunoblot analysis of 293 T cells expressing the indicated PTPN18 variants after the addition of 100 mg/ml cycloheximide (CHX). Band intensities at different exposure times were normalized the band intensities of ETS1 at 0 min. **E** Quantification of the data in D with ImageJ.

We observed that PTPN18 significantly decreased the expression of ETS1 (Fig. 4A; Fig. S5A). PTPN18 also regulated the phosphorylation of ETS1 by SRC at Y283, which prevents recognition by the E3 ubiquitin ligase COP1 (Fig. 4B; Fig. S5B) [42]. This effect was specific to the nucleus because of the nuclear localization of ETS1 in breast cancer cells (Fig. 4C). Consistent with these results, relative to the inactive mutant PTPN18 C229S or cytoplasmic PTPN18 (mC-NLS and mPY-NLS mutants), PTPN18 WT grossly destabilized ETS1 (Fig. 4D, E). These results collectively showed the differential effects of nuclear PTPN18 on spatiotemporal regulation.

### Nuclear PTPN18 inhibits EMT, TGFβ signaling, and motility in breast cancer cell lines

**Fig. 5:**
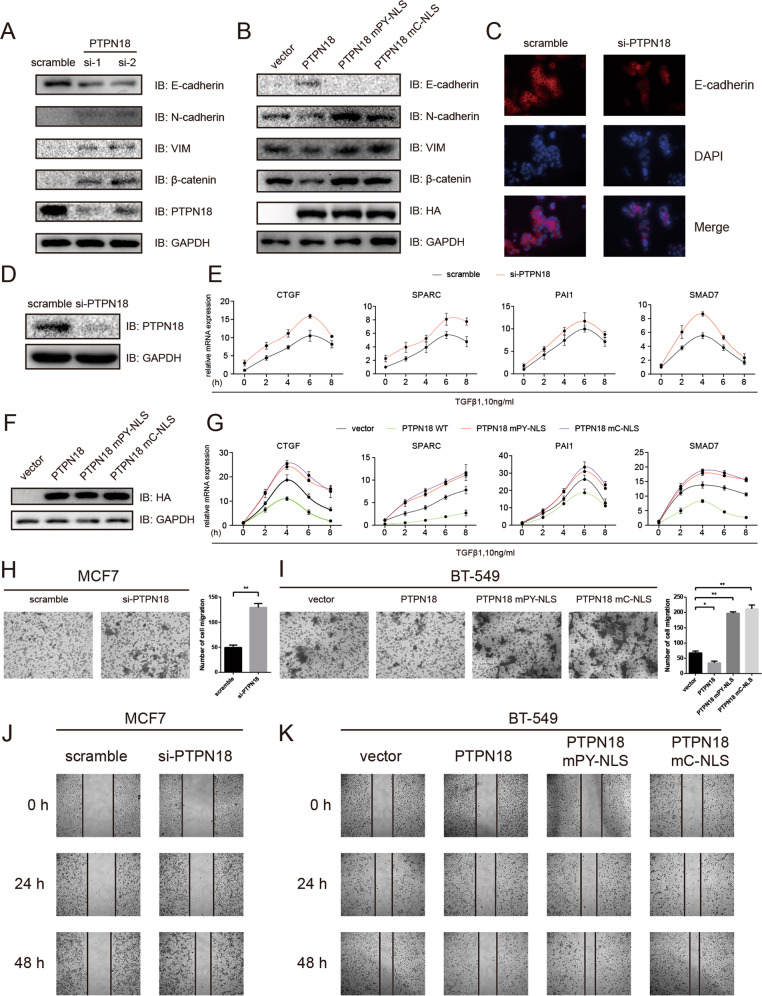
Nuclear PTPN18 suppresses EMT, TGF-β signaling, and motility. **A** Immunoblot analysis of PTPN18 and various EMT markers in MCF7 cells treated with PTPN18 siRNAs. **B** Immunoblot analysis of PTPN18 and various EMT markers in BT-549 cells after ectopic expression of PTPN18 derivatives. **C** Immunofluorescence of E-cadherin in MCF7 cells treated with PTPN18 siRNA. **D** Representative immunoblots showing the effects of PTPN18 knockdown in MCF7 cells. **E** qRT-PCR analysis of TGF-β downstream genes at the indicated time points in cells treated with TGFβ1 (10 ng/ml) and PTPN18 knockdown. **F** Representative immunoblots showing the expression of ectopic PTPN18 derivatives in BT-549 cells. **G** qRT-PCR analysis of TGF-β downstream genes in cells treated with TGFβ1 (10 ng/ml) and transfected with ectopic PTPN18 derivatives. **H**, **I** Representative images of the Transwell invasion assays of MCF7 cells with PTPN18 knockdown and BT-549 cells with ectopic expression of PTPN18 derivatives, respectively. The cell number was determined in six randomly acquired images. **J, K** Wound healing assays of MCF7 cells with PTPN18 knockdown and BT-549 cells with ectopic expression of PTPN18, respectively.

To acquire the ability to migrate, carcinoma cells almost invariably undergo the EMT process [5, 6]. Indeed, significant correlations between PTPN18 and EMT-related genes were observed in breast cancer cell lines (Fig. S3E–G). Knockdown of PTPN18 enhanced mesenchymal traits in MCF7 and T-47D cells (Fig. 5A, B; Fig S6A, B). Conversely, overexpression of PTPN18 WT but not cytoplasmic PTPN18 (mC-NLS and mPY-NLS) suppressed mesenchymal traits in BT-549 and MDA-MB-231 cells (Fig. 5C; Fig. S6C), indicating that nuclear PTPN18 plays a critical role in the regulation of EMT. Since TGFβ is a crucial regulator of EMT, we then tested TGFβ signaling by qRT-PCR analysis of a panel of genes that are involved in driving breast cancer metastasis to the bone or lung [43, 44] in the presence or absence of TGFβ1 (Fig. 5D–G; Fig. S6D–G). As expected, nuclear PTPN18 inhibited the expression of TGFβ1-regulated genes in breast cancer cells, and the suppressive role of nuclear PTPN18 in cell migration was validated by Transwell assays (Fig. 5H, I; Fig. S6H, I) and wound healing assays (Fig. 5J, K; Fig. S6J, K) in multiple breast cancer cell lines.

In order to fully reflect the whole pathway, we applied the MVP EA/TA mutant to prevent PTPN18 nuclear import and confirmed the role of nuclear PTPN18 in regulating breast cancer metastasis. We observed that MVP EA/TA could regulate the phosphorylation and degradation of ETS1 through PTPN18 (Fig. S7A, B). Moreover, we found that expression of ectopic MVP EA/TA, which removes the inhibition of downstream genes by nuclear PTPN18, significantly enhanced the mesenchymal traits and promoted the motility of MCF7 cells (Fig. S7C–F).

### Nuclear PTPN18 inhibits lung metastasis in nude mice

**Fig. 6:**
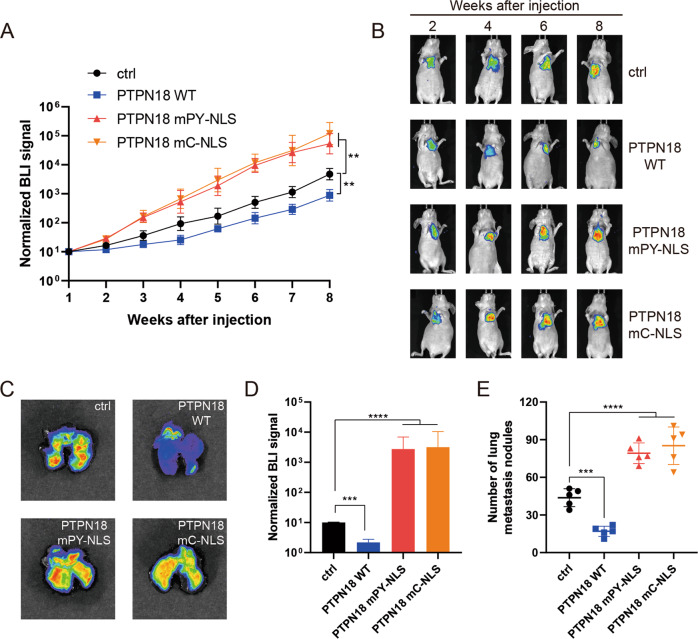
Nuclear PTPN18 inhibits lung metastasis in mouse model. **A** Normalized bioluminescence signals of lung metastases in mice injected intravenously with MDA-MB-231 cells with overexpressing PTPN18 WT or cytoplasmic PTPN18. **P < 0.01. **B** Representative bioluminescence image of mice in each experimental group at the indicated time points. **C** Representative bioluminescence image of lung metastatic nodules. **D** Normalized bioluminescence signals in lung metastatic nodules from mice. ***P < 0.001; ****P < 0.0001. **E** Quantification of lung metastatic nodules from mice. ***P < 0.001; ****P < 0.0001.

To estimate the effect of nuclear PTPN18 on lung metastasis, MDA-MB-231 cells overexpressing luciferase-labeled PTPN18 WT- and cytoplasmic PTPN18 were injected intravenously into nude mice, and the mice were subjected to bioluminescence imaging. PTPN18 WT cells but not cytoplasmic PTPN18 cells exhibited a reduced lung metastasis ability even at early time points (Fig. 6A, B), implying that PTPN18 may negatively affect cell extravasation and/or early seeding of lung metastases. Continued bioluminescence monitoring revealed a further reduction in metastatic outgrowth in the lungs of animals injected with PTPN18 WT-overexpressing cells (Fig. 6A, B). We further observed weaker bioluminescence signals in lung metastatic nodules and a decrease in the number of metastatic lesions produced in mice injected with PTPN18 WT cells compared to control and cytoplasmic PTPN18 cells (Fig. 6C–E). Together, these data implied that nuclear PTPN18 but not cytoplasmic PTPN18 antagonizes TGFβ signaling-regulated EMT and reduces the metastatic ability of breast cancer cells.

## Discussion

The function of PTPN18 in cancer is seemingly controversial in the literature. Previous studies have revealed that PTPN18 prevents tumor progression in breast and hepatocellular cancers [21, 45] but promotes the development of colorectal and endometrial cancer [26, 46]. We found that PTPN18 was localized in both the nucleus and cytoplasm in breast cancer rather than accumulated exclusively in the cytoplasm, as found in colorectal and endometrial cancers [26]. In addition, nuclear PTPN18 inhibited TGFβ signaling-regulated EMT and reduced the metastatic ability of breast cancer cells. Thus, we speculated that the subcellular localization of PTPN18 may be one of the reasons for its contrasting role in different cancers.

The localization of PTPN18 in the nucleus suggested the existence of a trafficking system responsible for its translocation from the cytoplasm to the nucleus. In this work, we delineated for the first time the nuclear import mechanism of PTPN18, identifying the NLS sequence and transporters responsible for its nuclear localization. Ectopic expression of a series of truncation and point mutant derivatives of PTPN18 indicated that the canonical NLS and the PY-NLS are indispensable and adequate for driving nuclear import of PTPN18. The first step of nucleocytoplasmic shuttling occurs when an importin distinguishes between its cargos and other proteins by the NLSs. The best-elucidated transport signal is the canonical NLS for nuclear protein import, which comprises either one stretch (monopartite) or two (bipartite) stretches of a basic sequence [47], for example, the monopartite NLS in SV40 large T antigen (PKKKRRV) and the bipartite NLS in nucleoplasmin (KRPAATKKAGQAKKKK). Structural [48] and thermodynamic [49] experiments have confirmed the critical requirements for a canonical NLS, which is defined as a loose consensus sequence of K(K/R)X(K/R). The canonical NLS of PTPN18 is similar to this sequence. We also found that mutations in the K/R sites in the canonical NLS of PTPN18 prevented its nuclear accumulation in living cells. In addition, PTPN18 contains a conserved PY-NLS that consists of the consensus sequence R/KX_2–5_PY in its N-terminal region [50]. Consistent with our finding that the PY-NLS/importin β2 nuclear transport system regulates PTPN18 nuclear localization in the presence of MVP, we established that knockdown of importin β2 or MVP prevents PTPN18 nuclear accumulation in breast cancer. PTPN18 was pulled down by importin β2 rather than other importins in our IP experiments. That the PY-NLS conferred the ability for recognition by importin β2 was shown in the pull-down assay. The protein truncation experiments also showed that E146 and T150 in MVP are responsible for the interaction with the C-NLS of PTPN18, and this finding was further validated in vitro by a pull-down assay. Overall, these results support the idea that MVP and importin β2 cooperatively promote PTPN18 translocation from the cytoplasm to the nucleus in breast cancer cells.

**Fig. 7:**
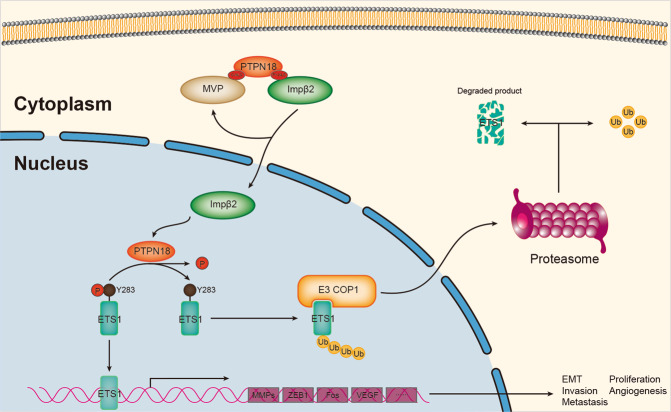
Schematic diagram showing MVP- and importin β2-mediated PTPN18 nuclear import and ETS1 stabilization regulated by nuclear PTPN18. PTPN18 interacts with MVP through cNLS, and interacts with importin β2 through PY-NLS, which synergistically promote the translocation of PTPN18 into the nucleus. PTPN18 is carried into the nucleus by importin β2. Nuclear PTPN18 can specifically recognize the substrate ETS1 and dephosphorylate its Y283 site, which promotes the degradation of ETS1. Nuclear PTPN18 inhibits breast cancer cell EMT, invasion and metastasis by regulating the ETS1.

ETS1 is a nuclear protein and functions as a transcriptional activator. Previous studies have shown that ETS1 is an effector of the TGFβ signaling pathway and plays an essential role in EMT, invasion, and angiogenesis during cancer metastasis [41, 51]. In the breast epithelium, ETS1 is primarily expressed in the triple-negative subtype [41], and its expression is inversely correlated with the expression of PTPN18. Intriguingly, a recent investigation showed that phosphorylation of S276 and S282 promotes the interaction between ETS1 and COP1, which induced ETS1 protein ubiquitination and degradation. However, phosphorylation of Y283 by SRC antagonizes these effects [42]. Adding to our finding that ectopic expression of PTPN18 resulted in dephosphorylation of ETS1 and promoted its degradation, we further delineated the role of nuclear PTPN18 in regulating breast cancer metastasis by gain/loss-of-function experiments. PTPN18 WT decreased ETS1 stability and inhibited breast cancer metastasis, but two cytoplasmic PTPN18 mutants, mC-NLS and mPY-NLS, stabilized ETS1 and restored the metastatic ability by spatiotemporal regulation. Given the downregulated expression of PTPN18 in triple-negative breast cancer cells, it can also be inferred that the degradation of ETS1 regulated by PTPN18 may be one of the reasons that ETS1 is only expressed in triple-negative breast cancer cells. Our extensive analysis of PTPN18 in EMT and metastasis indicated that nuclear PTPN18 but not cytoplasmic PTPN18 antagonized TGFβ signaling-regulated EMT and reduced the metastatic ability of breast cancer cells through regulation of ETS1 phosphorylation in breast cancer (Fig. 7).

Collectively, our analyses, for the first time, delineate the nuclear import mechanism of PTPN18, and we have concluded that nuclear PTPN18, through direct negative regulation of ETS1, functions as a master regulator of epithelial cell fate. PTPN18 acts as a suppressor of EMT and cancer metastasis in pathological contexts. Notably, downregulation of PTPN18 has frequently been detected in metastatic breast cancer tissues. Therefore, this event may represent one of the driving forces for early-stage breast tumor cells to undergo EMT and subsequent metastatic progression, thus highlighting the role of PTPN18 as a potential target for early therapeutic intervention in breast cancer progression.

## Materials and methods

### Human tissue sample collections

Human breast cancer samples, including primary and metastatic tumors, were collected from patients undergoing clinical surgery in the First Affiliated Hospital of Bengbu Medical College. Tissue samples were stored at −80 °C before use. The ethics committee of Bengbu Medical College approved experiments involving human specimens.

### Cell culture and transfections

The cell lines BT-549, MCF7, T-47D, Hs 578 T, HCC1937, MDA-MB-231, and 293 T were obtained from the Shanghai Institute of Biochemistry and Cell Biology, Chinese Academy of Sciences (Shanghai, China). BT-549, MCF7, T-47D, Hs 578 T, HCC1937, and 293 T cells were cultured in Dulbecco’s modified Eagle’s medium (DMEM; HyClone, Waltham, MA, USA) supplemented with 10% fetal bovine serum (FBS; HyClone, Waltham, MA, USA) and 1% penicillin/streptomycin (P/S) at 37 °C in a humidified incubator with 5% CO_2_. MDA-MB-231 cells cultured in Leibovitz’s L-15 Medium (Gibco, Carlsbad, CA, USA) supplemented with 10% FBS and 1% P/S at 37 °C in a humidified incubator. Following the manufacturer’s instructions, Lipofectamine 3000 (Invitrogen, Carlsbad, CA, USA) was used to transiently transfect plasmids and siRNAs. The sequences of the siRNAs used in this study are listed in Supplementary Table 1.

### Immunoprecipitation assay

Fresh cells were washed with PBS twice and lysed in cold RIPA lysis buffer with protease inhibitor cocktail (Roche, Mannheim, Germany) and the phosphatase inhibitor cocktail PhosSTOP (Roche, Mannheim, Germany) 48 h after transfection of target plasmids or without transfection if targeting endogenous proteins. Cell lysates were centrifuged at 12,000 × g for 15 min to remove cell debris and incubated with the appropriate primary antibodies at 4 °C overnight with gentle agitation. The next day, the lysates were incubated with protein A/G agarose beads (Beyotime, Shanghai, China) at 4 °C for 2 h. After incubation, the beads were washed with cold lysis buffer three times and boiled with 1 × SDS loading buffer for 7 min. Proteins in the supernatants were separated by SDS-PAGE prior to western blot analysis.

### Western blot analysis

After extracting total protein from cells or tissues, equivalent amounts of denatured proteins from each sample were separated using SDS-PAGE and then transferred to PVDF membranes. The membranes were incubated overnight with targeted primary antibodies at 4 °C before blocking with 5% BSA in TBST at room temperature for 1 h. The membranes were washed with TBST three times for 5 min each, prior to incubation with secondary antibodies at room temperature for 1 h. The membranes were scanned, visualized, and analyzed using a ChemiDoc system (BioRad, Hercules, CA, USA) and Image Lab software (BioRad, Hercules, CA, USA). Antibody information is listed in Supplementary Table 2.

### Confocal immunofluorescence microscopy

Cells were cultured on coverslips in 24-well plates. Forty-eight hours after transfection, the cells were fixed for 30 min using 4% paraformaldehyde solution, and then permeabilized using 0.1% Triton X-100 in PBS for 30 min at room temperature. Nonspecific binding sites were blocked for 1 h with 5% BSA in TBST. After that, the cells were incubated with primary antibodies at 4 °C overnight and then incubated with secondary antibodies for 2 h at room temperature. After counterstaining with DAPI (Beyotime, Shanghai, China) for 5 min, the coverslips were sealed with antifade mounting medium (Beyotime, Shanghai, China) and observed under a Leica SP5 confocal laser scanning microscope (Leica Microsystems, Wetzlar, Germany).

### Xenograft model of breast cancer lung metastasis

Six-week-old female BALB/c mice were used for the lung metastasis experiments. MDA-MB-231 cells (1 × 10^6^) in 200 μl of PBS solution were injected into the lateral tail vein of nude mice. After intraperitoneal injection of luciferase substrate and isoflurane inhalation anesthesia, bioluminescence imaging was performed weekly for monitoring. The mice were killed after 8 weeks. Metastatic nodules on the surface of the lungs were counted under a dissecting microscope. Signals were normalized to the initial post-injection signals on Day 0. All mice were housed and handled following protocols approved by the Animal Care and Use Committee of Northeastern University.

## Supplementary information

Supplementary methods

Supplementary figure 1

Supplementary figure 2

Supplementary figure 3

Supplementary figure 4

Supplementary figure 5

Supplementary figure 6

Supplementary figure 7

Supplementary figure legends

Supplementary Tables 1

Supplementary Tables 2

Original Data File

Reproducibility checklist

## Data Availability

All patient data used in this work can be acquired from the Gene Expression Omnibus (GEO; https://www.ncbi.nlm.nih.gov/geo/) under the accession number GSE52604, GSE57968, GSE32489, GSE20565, GSE124647, GSE14020, GSE20685 and GSE5327, and the GDC portal (TCGA; https://portal.gdc.cancer.gov/). The omics data of cell lines were acquired from the Broad Institute Cancer Cell Line Encyclopedia (CCLE, https://portals.broadinstitute.org/ccle).
