# Supplementary material for: Nuclear import of PTPN18 inhibits breast cancer metastasis mediated by MVP and importin β2
Source: Cell Death Dis. 2022 Aug 18;13(8):720. doi: 10.1038/s41419-022-05167-z (PMC9388692; doi:10.1038/s41419-022-05167-z)
Supplement: Supplementary file 1 — Supplementary methods [file 41419_2022_5167_MOESM1_ESM.docx]

SUPPLEMENTARY MATERIALS AND METHODS

RNA isolation and quantitative real-time PCR

Total RNA was extracted using TRIzol reagent (Invitrogen, Carlsbad, CA, USA). According to the manufacturer's instruction, cDNA synthesis and qRT-PCR were performed using GoScript reverse transcriptase (Promega) and Master Mix (Promega). Data was collected using the CFX96 Touch real-time PCR detection system (BioRad, Hercules, CA, USA) and then calculated by the 2^-ΔΔCq^ method with normalizing GAPDH as the reference gene. The primers were listed in Supplementary Table 1.

Protein pull-down assay

Plasmids expressing target proteins were transformed in *E. coil* and treated with 0.3 mM IPTG for 12 h at 25 ℃. Cells were suspended in cold PBS and homogenized by sonication. After purification with Glutathione-Sepharose 4B beads (Fisher Scientific, Carlsbad, CA, USA), equivalent amounts of the purified GST fusion proteins were incubated with His-tagged protein for 8 h at 4 ℃. The Sepharose was washed with cold lysis buffer three times and then boiled with 1 × SDS loading buffer for 7 min. The supernatants were resolved by SDS-PAGE followed by western blot analysis.

IP and LC-MS/MS

IP products were generated as described above. After in-gel tryptic digestion, the tryptic peptides were dissolved in 0.1% formic acid (Fluka) directly loaded onto a reversed-phase pre-column (Acclaim PepMap 100, Fisher Scientific, Carlsbad, CA, USA). Peptide separation was performed using a reversed-phase analytical column (Acclaim PepMap RSLC, Fisher Scientific, Carlsbad, CA, USA). The gradient was comprised of an increase from 6% to 25% solvent B (0.1% formic acid in 98% acetonitrile) over 16 min, 25% to 40% over 6 min and climbing to 80% in 4 min, then holding at 80% for the last 4 min, all at a constant flow rate of 320 nl/min on an EASY-nLC 1000 UPLC system. The peptides were subjected to NSI source followed by tandem mass spectrometry (MS/MS) in Q Exactive^TM^ (Fisher Scientific, Carlsbad, CA, USA) coupled online to the UPLC. Intact peptides were detected in the orbitrap at a resolution of 70,000. Peptides were selected for MS/MS using NCE setting as 28; ion fragments were detected in the orbitrap at a resolution of 17,500. A data-dependent procedure alternated between one MS scan followed by 20 MS/MS scans was applied for the top 20 precursor ions above a threshold ion count of 5E3 in the MS survey scan with 15 s dynamic exclusion. The electrospray voltage applied was 2.0 kV. Automatic gain control (AGC) was used to prevent overfilling of the orbitrap; 5E4 ions were accumulated for the generation of MS/MS spectra. For MS scans, the m/z scan range was 350 to 1800. The resulting MS/MS data were processed using the Mascot search engine (v. 2.3.0). Tandem mass spectra were searched against the SwissProt Homo sapiens sequence. Trypsin/P was specified as a cleavage enzyme allowing up to 2 missing cleavages. The mass error was set to 10 ppm for precursor ions and 0.02 Da for-fragment ions. Carbamidomethyl on Cys was specified as fixed modification and oxidation on Met, acetylation on Protein N term were specified as variable modifications. Peptide ion score was set ≥ 20.

Immunohistochemistry staining

Immerse the target tissue in 4% paraformaldehyde overnight at room temperature. Then placed in the embedding box, and rinsed with running water for 2-4 h. The tissues were dehydrated in 65%, 75%, 85%, 95%, 95%, 100%, and 100% graded ethanol for 1 h each time. The tissue was placed in xylene twice, each for 15 min. At this time, the tissue should be clear by visual inspection. The transparent tissue was placed in soft wax and hard wax for 1 h to remove residual xylene. Place the tissue in paraffin, and take it out for later use after it has completely solidified. Use a microtome for slicing, and place the slices in water. After fully unfolding, place them on glass slides and dry them for later use. Place the slices into xylene twice for 15 min; 100%, 100%, 95%, 90%, 85%, 75%, 65% gradient ethanol and water, 3-5 min each time. The sections were placed in the incubation box and washed three times with PBS for 5 min each time. After that, the sections were immersed in 0.01 M citrate buffer, heated to boiling in a microwave oven, cooled at room temperature (about 30-60 min), and washed three times with PBS for 5 min each time. Shake off the residual PBS, use an oil pen to delineate the desired area, add endogenous peroxidase, incubate at room temperature for 10 min, and then wash with PBS three times, 5 min each time. The residual PBS was shaken off. Non-specific staining was added to the block, incubated at room temperature for 10 min, and washed three times with PBS for 5 min each time. Add primary antibody and incubate overnight at 4°C. The next day, washed three times with PBS for 5 min each time. The PBS was shaken off, biotin-labeled goat anti-rabbit IgG polymer was added, incubated at room temperature for 10 min, and then washed three times with PBS, 5 min each time. Incubate for 10 min at room temperature and wash three times with PBS for 5 min each. PBS was removed, and DAB was added for color development. Rinse with running water and counterstain with hematoxylin. Neutral gum was added dropwise to the section and covered with a coverslip. Read under the microscope and take pictures.

Wound healing and Transwell assays

Cells were cultured in 6-well plates for wound healing assay until 70% confluency. The sterile plastic pipette tip was used to generate a scratch wound in the center of the cell monolayer. Cell debris was removed by PBS washing. Then, cells were cultured in a serum-free medium. An inverted microscope photographed the wound.

For the Transwell assay, 24-well plates were used. A total 1 × 10^5^ cells per well were resuspended in 0.4 ml serum-free medium and seeded into the upper chamber. 0.6 ml medium with 20% FBS was added to the lower chamber as the chemoattractant. 24 h later, cells migrating to the lower chamber were fixed with 4% paraformaldehyde solution for 30 min at room temperature, then stained with 0.2% crystal violet for 20 min at room temperature, and counted in three randomly selected fields under an inverted microscope.

Bioinformatics analysis

For analysis of PTPN18 interactome, CRAPome was utilized to remove background contaminants from bona fide interaction proteins [1]. To evaluate the relationship strength between the target protein and its partners by considering the function and location of proteins, we used the GOSemSim R package to assess the semantic similarities among interactome proteins in molecular function and cellular component [2]. This step was adjusted by the Wang method, which is more accurate and unbiased by considering the GO topological structure [3].

To evaluate PTPN18 expression in human breast cancer patients, we combined several datasets using Combat [4] as defined below. The normal, adjacent, and primary tissue of breast cancer were extracted from the gene expression profiles of the TCGA project, and the Genotype-Tissue Expression (GTEx) project that is re-computed by the UCSC Xena project depended on a defined pipeline. Normal tissue was acquired from patients in the GTEx project; Tumor adjacent and primary tissue were acquired from patients in the TCGA-BRCA cohort. Metastatic samples of breast cancer distributed in different tissue were downloaded from the Gene Expression Omnibus (GEO) database under the accession numbers GSE52604, GSE57968, GSE32489, GSE20565, GSE124647, and GSE14020.

For evaluation of PTPN18 expression in human cell lines, gene expression profiles were acquired from the Broad Institute Cancer Cell Line Encyclopedia (CCLE) [5].

For predicting the prognosis of PTPN18 in breast cancers, clinical data of TCGA-BRCA were acquired using TCGAbiolinks [6]. Moreover, GSE20685 and GSE5327 were used to assess the distant metastasis-free survival and lung metastasis-free survival, respectively.

Statistical analysis

All experiments were conducted at least three biological replicates. The data were displayed as mean ± S.D. or mean ± S.E.M. as indicated in the figures. To compare central tendencies, normally distributed unpaired two-sided Student’s t-tests analyzed data sets under the assumption of equal variance; non-normally distributed data sets were analyzed by non-parametric Mann-Whitney U-tests. Differences were considered statistically significant when *P* < 0.05.

REFERENCES

1. Mellacheruvu D, Wright Z, Couzens AL, Lambert JP, St-Denis NA, Li T*, et al.* The CRAPome: a contaminant repository for affinity purification-mass spectrometry data. Nature methods. 2013;**10:**730-736.

2. Yu G, Li F, Qin Y, Bo X, Wu Y, Wang S. GOSemSim: an R package for measuring semantic similarity among GO terms and gene products. Bioinformatics. 2010;**26:**976-978.

3. Wang JZ, Du Z, Payattakool R, Yu PS, Chen CF. A new method to measure the semantic similarity of GO terms. Bioinformatics. 2007;**23:**1274-1281.

4. Johnson WE, Li C, Rabinovic A. Adjusting batch effects in microarray expression data using empirical Bayes methods. Biostatistics. 2007;**8:**118-127.

5. Barretina J, Caponigro G, Stransky N, Venkatesan K, Margolin AA, Kim S*, et al.* The Cancer Cell Line Encyclopedia enables predictive modelling of anticancer drug sensitivity. Nature. 2012;**483:**603.

6. Colaprico A, Silva TC, Olsen C, Garofano L, Cava C, Garolini D*, et al.* TCGAbiolinks: an R/Bioconductor package for integrative analysis of TCGA data. Nucleic Acids Res. 2016;**44:**e71.
