# Supplementary figures and images for: Nuclear import of PTPN18 inhibits breast cancer metastasis mediated by MVP and importin β2
Source: Cell Death Dis. 2022 Aug 18;13(8):720. doi: 10.1038/s41419-022-05167-z (PMC9388692; doi:10.1038/s41419-022-05167-z)

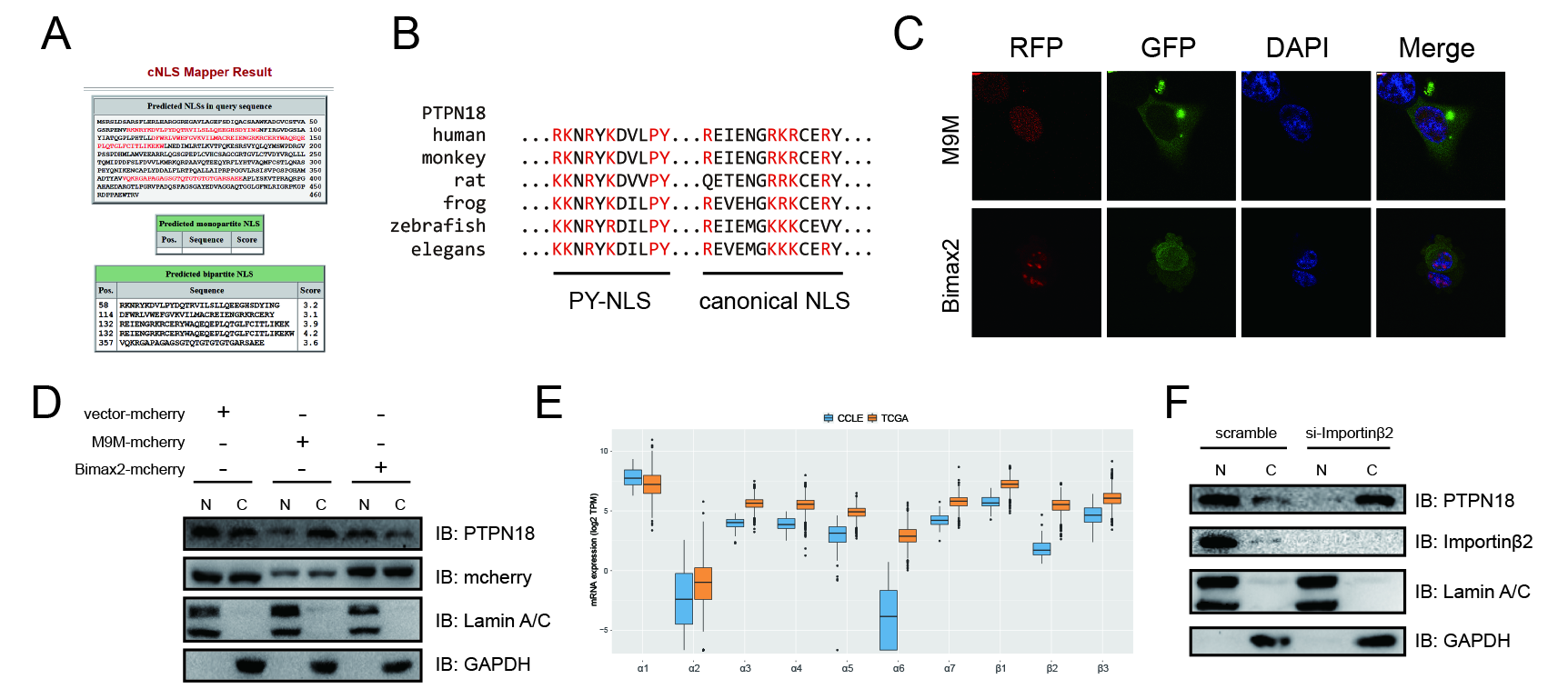

Supplement: Supplementary file 2 — Supplementary figure 1 [file 41419_2022_5167_MOESM2_ESM.tif]

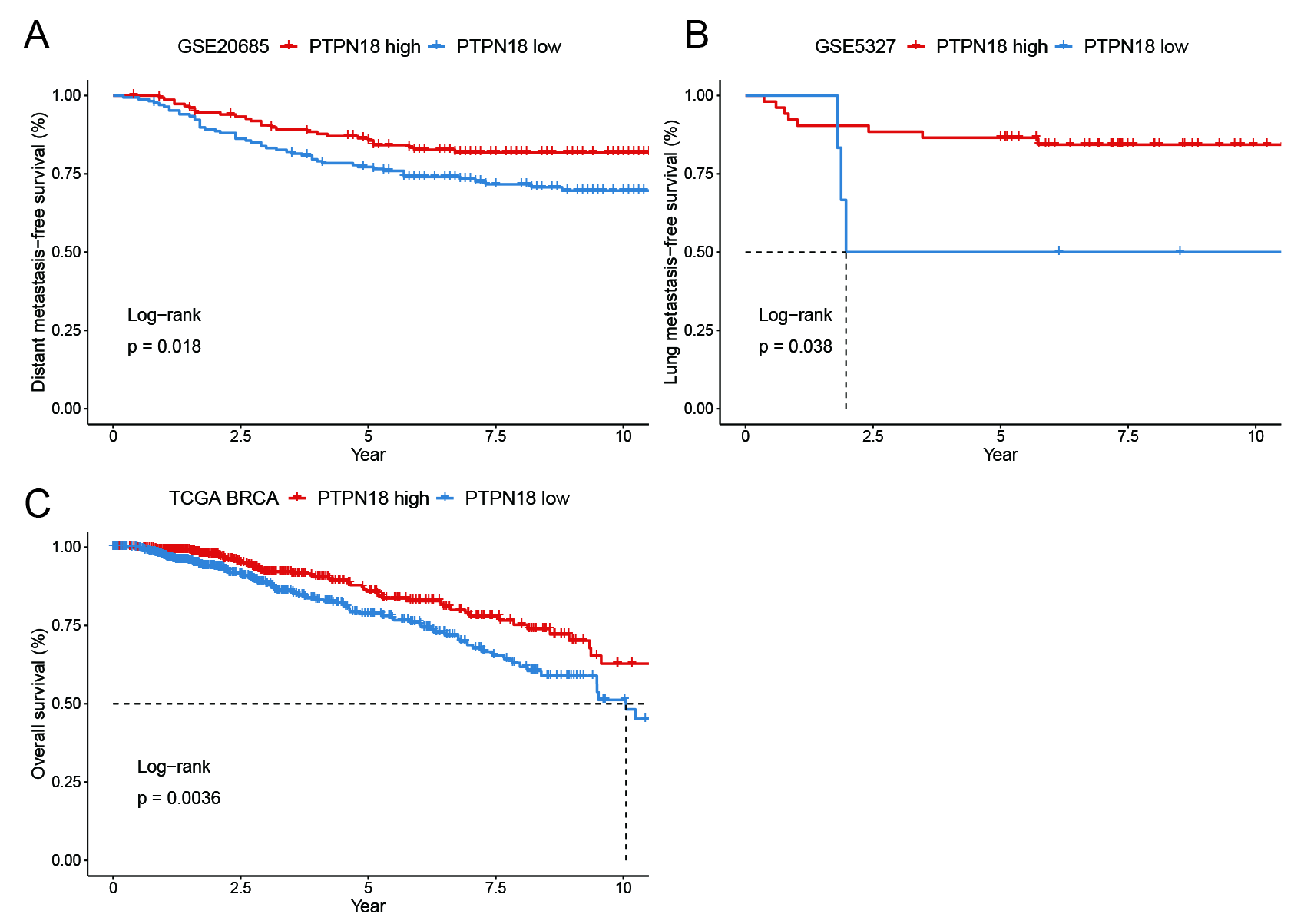

Supplement: Supplementary file 3 — Supplementary figure 2 [file 41419_2022_5167_MOESM3_ESM.tif]

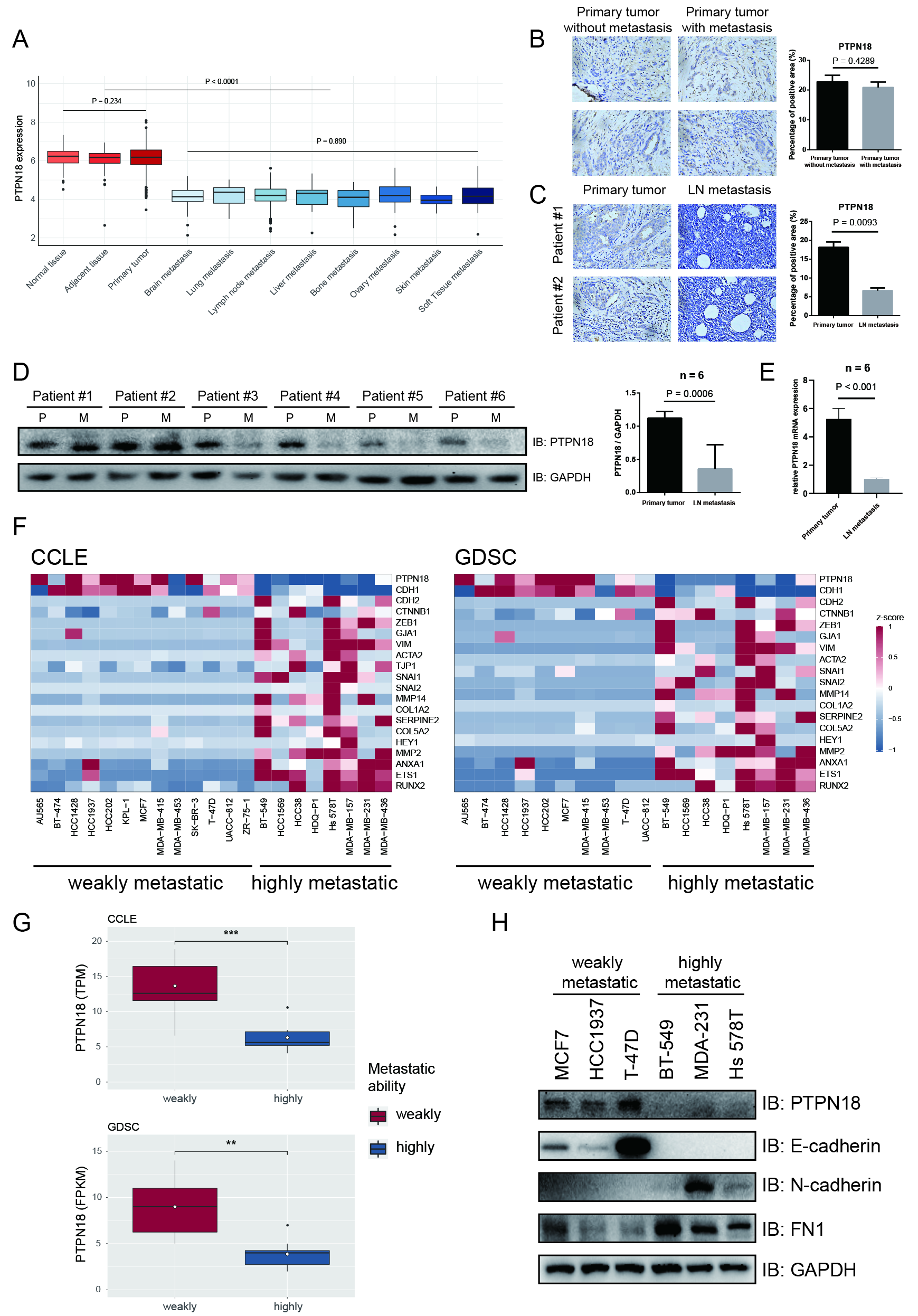

Supplement: Supplementary file 4 — Supplementary figure 3 [file 41419_2022_5167_MOESM4_ESM.tif]

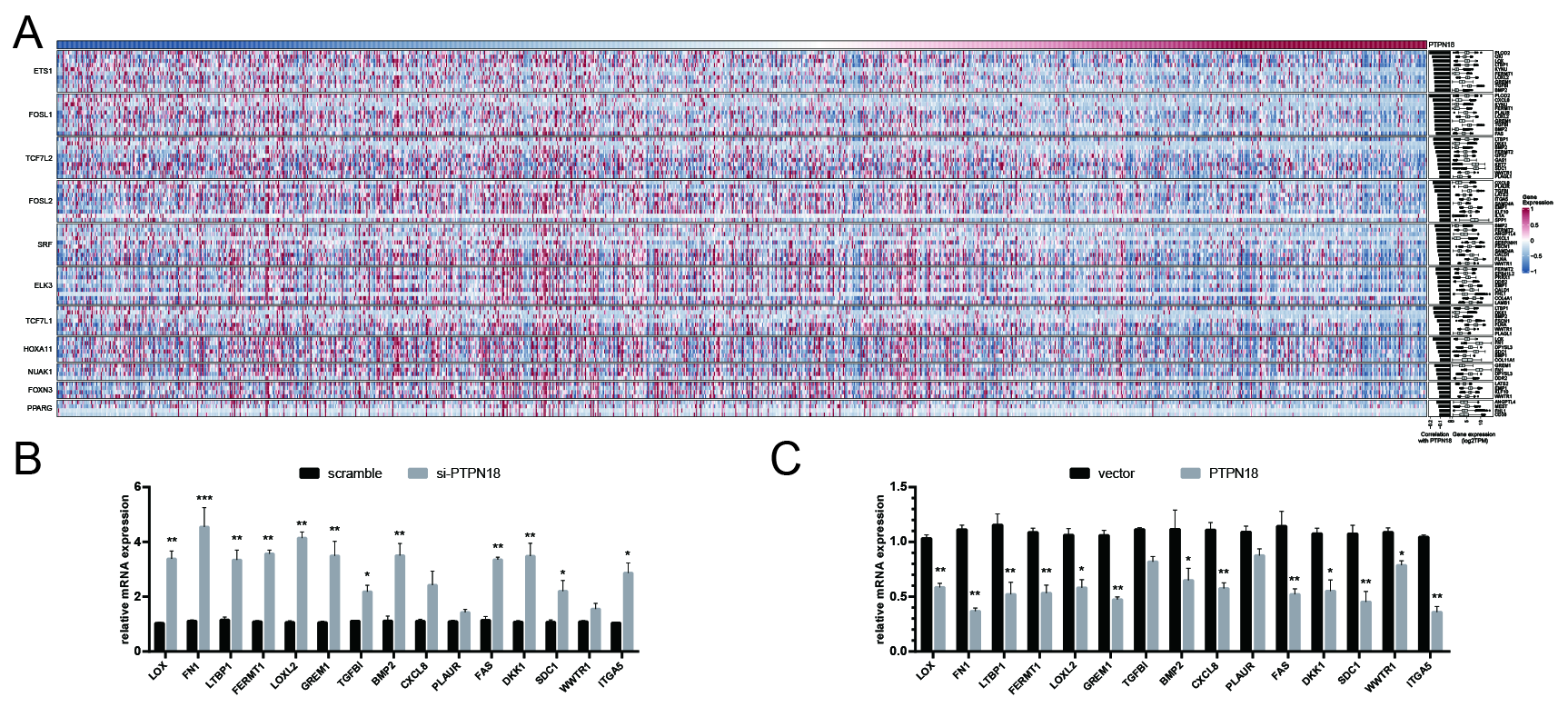

Supplement: Supplementary file 5 — Supplementary figure 4 [file 41419_2022_5167_MOESM5_ESM.tif]

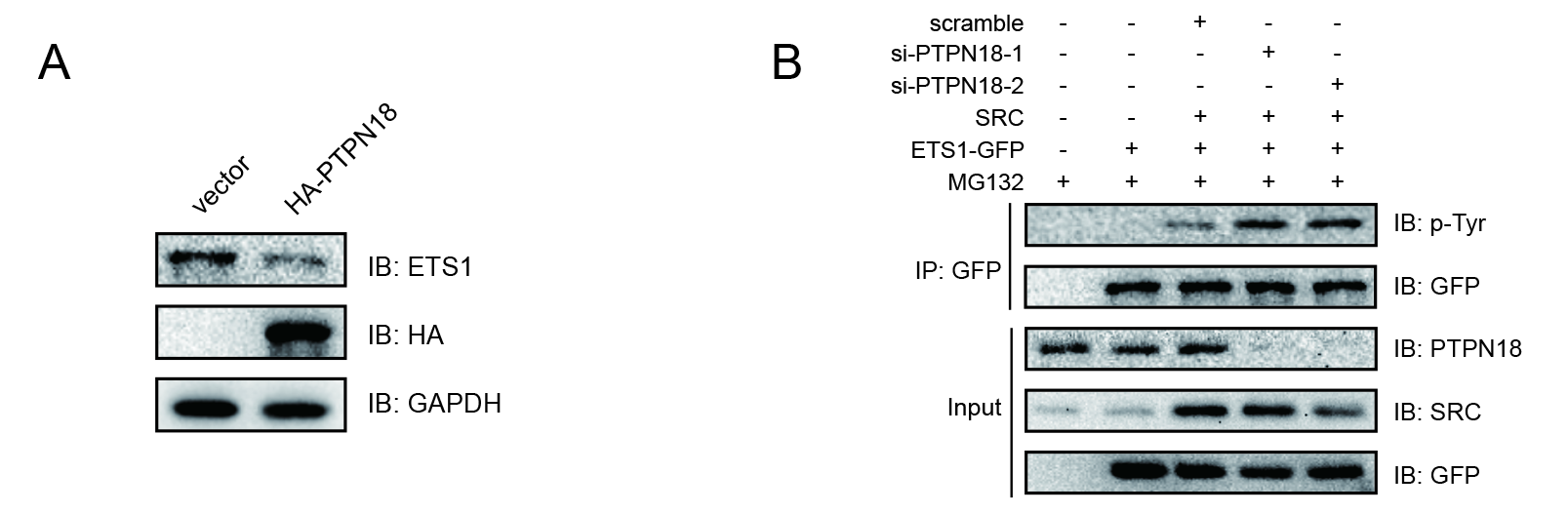

Supplement: Supplementary file 6 — Supplementary figure 5 [file 41419_2022_5167_MOESM6_ESM.tif]

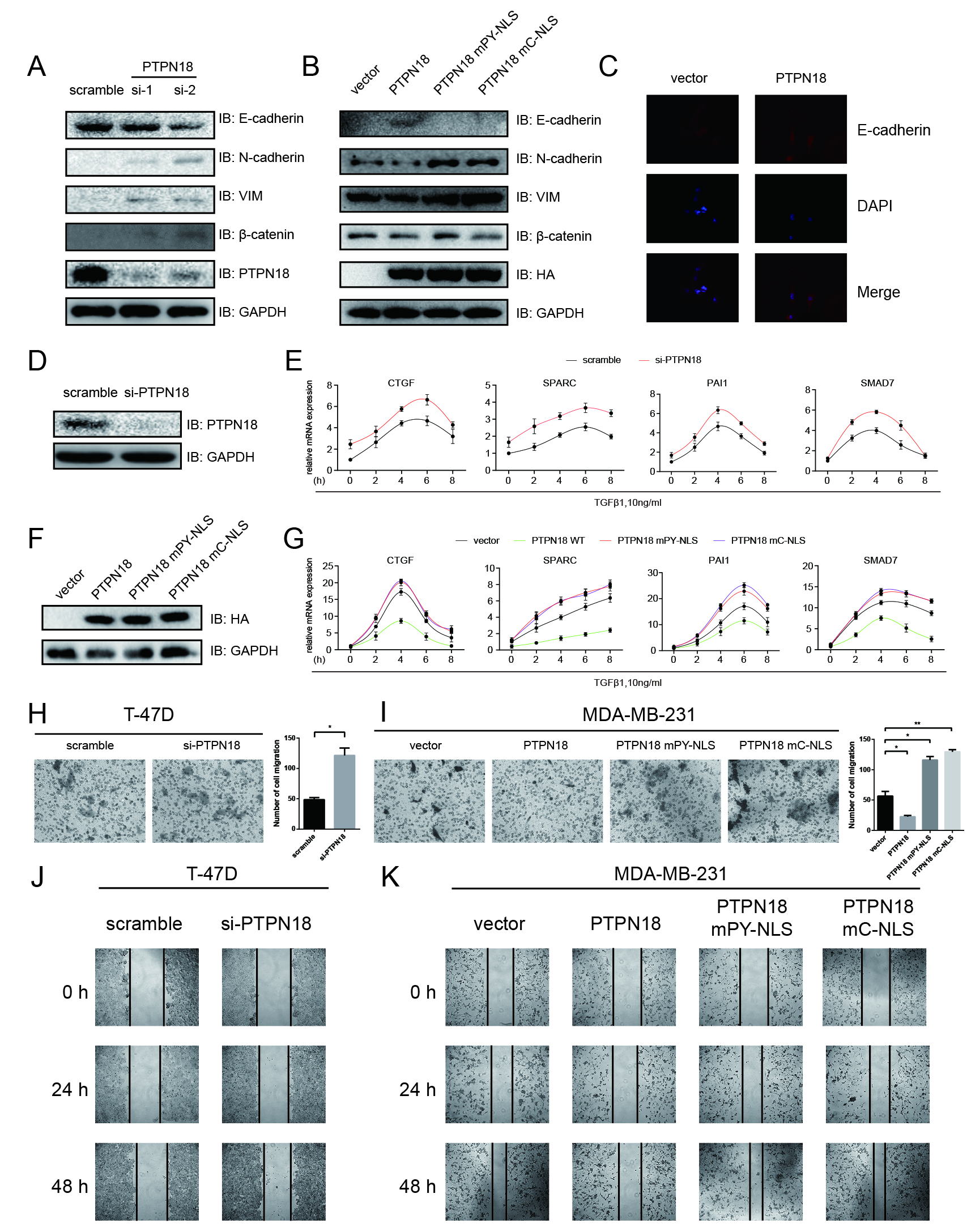

Supplement: Supplementary file 7 — Supplementary figure 6 [file 41419_2022_5167_MOESM7_ESM.tif]

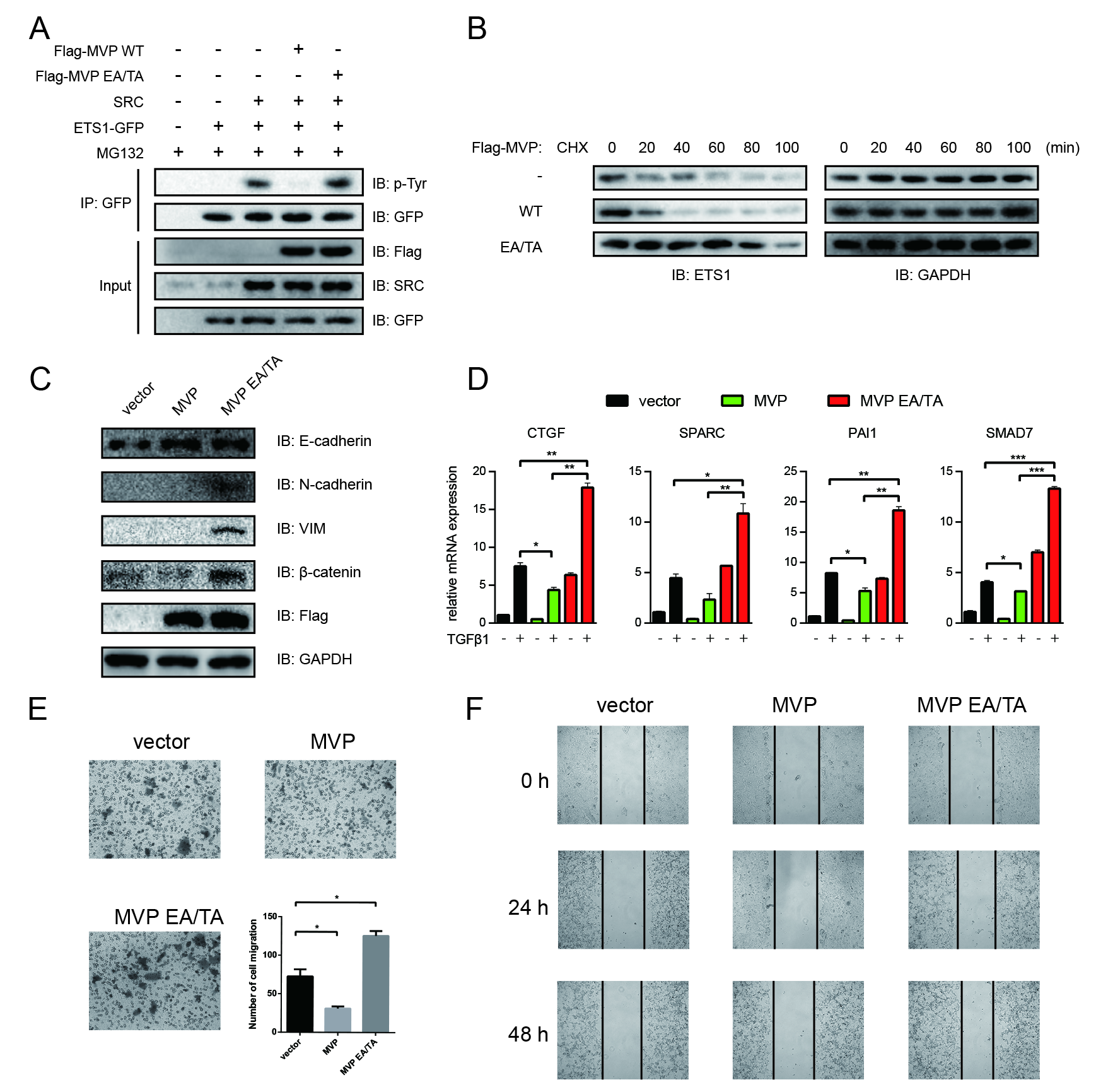

Supplement: Supplementary file 8 — Supplementary figure 7 [file 41419_2022_5167_MOESM8_ESM.tif]
