# Supplementary material for: Nuclear import of PTPN18 inhibits breast cancer metastasis mediated by MVP and importin β2
Source: Cell Death Dis. 2022 Aug 18;13(8):720. doi: 10.1038/s41419-022-05167-z (PMC9388692; doi:10.1038/s41419-022-05167-z)
Supplement: Supplementary file 9 — Supplementary figure legends [file 41419_2022_5167_MOESM9_ESM.docx]

SUPPLEMENTARY FIGURE LEGENDS

**Fig. S1 PTPN18 nuclear import is required for importin ꞵ2. A** Prediction of NLS in PTPN18 using cNLS mapper. **B** Residues conserved across species within PTPN18. **C-D** Affections of PTPN18 distribution by M9M and Bimax2 using immunofluorescence and immunoblot, respectively. **E** Expression distribution of importin family in human breast tissue (TCGA) and cell lines (CCLE). **F** Immunoblot of PTPN18 distribution after transient knockdown of importin β2.

**Fig. S2** **PTPN18 predicts** **a favorable prognosis of breast cancers. A** Distant metastasis-free survival of breast cancers was stratified by PTPN18 level in the GSE20685 dataset. **B** Lung metastasis-free survival of breast cancers based on PTPN18 level in GSE5327 dataset. **C** PTPN18 is associated with a favorable prognosis in the TCGA cohort.

**Fig. S3 PTPN18 is associated with breast cancer metastasis. A** PTPN18 expression pattern was analyzed in metastatic tumors using the combined datasets. **B** PTPN18 expression level was examined in primary breast cancer tissues with and without metastasis by immunohistochemistry (IHC). The quantification of IHC score was measured by Image-Pro Plus software. **C-E** PTPN18 mRNA and protein levels were examined between human primary breast tumor and lymph node metastasis tumor by IHC, western blot, and qPCR. P: primary tumor; M: metastatic tumor. The quantification of western blot was measured by Image J. **F** Heatmap demonstrates relative expression of PTPN18 and representative metastasis-related genes in various breast cancer cell lines grouped by metastatic ability. **G** Boxplot showing the statistic result of PTPN18 expression in two independent datasets, **P < 0.01, ***P < 0.001. **H** Immunoblot of PTPN18 expression and representative metastasis-related genes in breast cancer cell lines grouped by metastatic ability.

**Fig. S4 Prediction of potential substrates of PTPN18 in breast cancer. A** Heatmap showing the correlation between the expression of PTPN18 and downstream genes of potential substrates. **B** qRT-PCR analysis of target genes after knockdown PTPN18. **C** qRT-PCR analysis of target genes after ectopic expression of PTPN18.

**Fig. S5. PTPN18 decreases ETS1 expression and regulates its phosphorylation.** **A** Immunoblot analysis of ETS1 expression after PTPN18 overexpression. **B** Immunoblot analysis of ETS1 tyrosine pahosphorylation after PTPN18 knockdown. Cells were treated with 10 mM MG132 6 h before harvest.

**Fig. S6 Nuclear PTPN18 suppresses EMT, TGF-β signaling, and motility. A** Immunoblot of PTPN18 and various EMT markers in T-47D cells treated with PTPN18 siRNAs. **B** Immunoblot of PTPN18 and various EMT markers in MDA-MB-231 cells treated with ectopic expression of PTPN18 derivatives. **C** Immunofluorescence of E-cadherin in T-47D cells treated with PTPN18 siRNA. **D** Representative immunoblots showing knockdown PTPN18 expression in T-47D cells. **E** qRT-PCR analysis of TGF-β downstream genes at indicated time points in cells treated with TGFβ1 (10 ng/ml) and knockdown PTPN18. **F** Representative immunoblots showing ectopic PTPN18 derivatives expression in MDA-MB-231 cells. **G** qRT-PCR analysis of TGF-β downstream genes in cells treated with TGFβ1 (10 ng/ml) and ectopic PTPN18 derivatives. **H-I** Representative images of transwell invasion assay for knockdown PTPN18 in T-47D cells and ectopic PTPN18 derivatives in MDA-MB-231 cells, respectively. Cell number was counted in six randomly captured pictures. **J-K** Wound healing assay of knockdown PTPN18 in T-47D cells and ectopic PTPN18 in MDA-MB-231 cells, respectively.

**Fig. S7 MVP suppresses EMT, TGF-β signaling, and motility through interaction with PTPN18. A** Immunoblot analysis of ETS1 tyrosine phosphorylation after ectopic expression of MVP WT and EA/TA. Cells were treated with 10 mM MG132 6 h before harvesting. **B** Immunoblot analysis of 293T cells expressing the indicated MVP variants after the addition of 100 mg/ml cycloheximide (CHX). **C** Immunoblot of MVP and various EMT markers in MCF7 cells treated with ectopic expression of MVP derivatives. **D** qRT-PCR analysis of TGF-β downstream genes in cells treated with TGFβ1 (10 ng/ml) and ectopic MVP derivatives. **E** Representative images of transwell invasion assay for ectopic MVP derivatives in MCF7 cells. Cell number was counted in six randomly captured pictures. **F** Wound healing assay of ectopic MVP derivatives in MCF7 cells.
