# Supplementary material for: Nuclear import of PTPN18 inhibits breast cancer metastasis mediated by MVP and importin β2
Source: Cell Death Dis. 2022 Aug 18;13(8):720. doi: 10.1038/s41419-022-05167-z (PMC9388692; doi:10.1038/s41419-022-05167-z)
Supplement: Supplementary file 12 — Original Data File [file 41419_2022_5167_MOESM12_ESM.pdf]

Fig. 1C

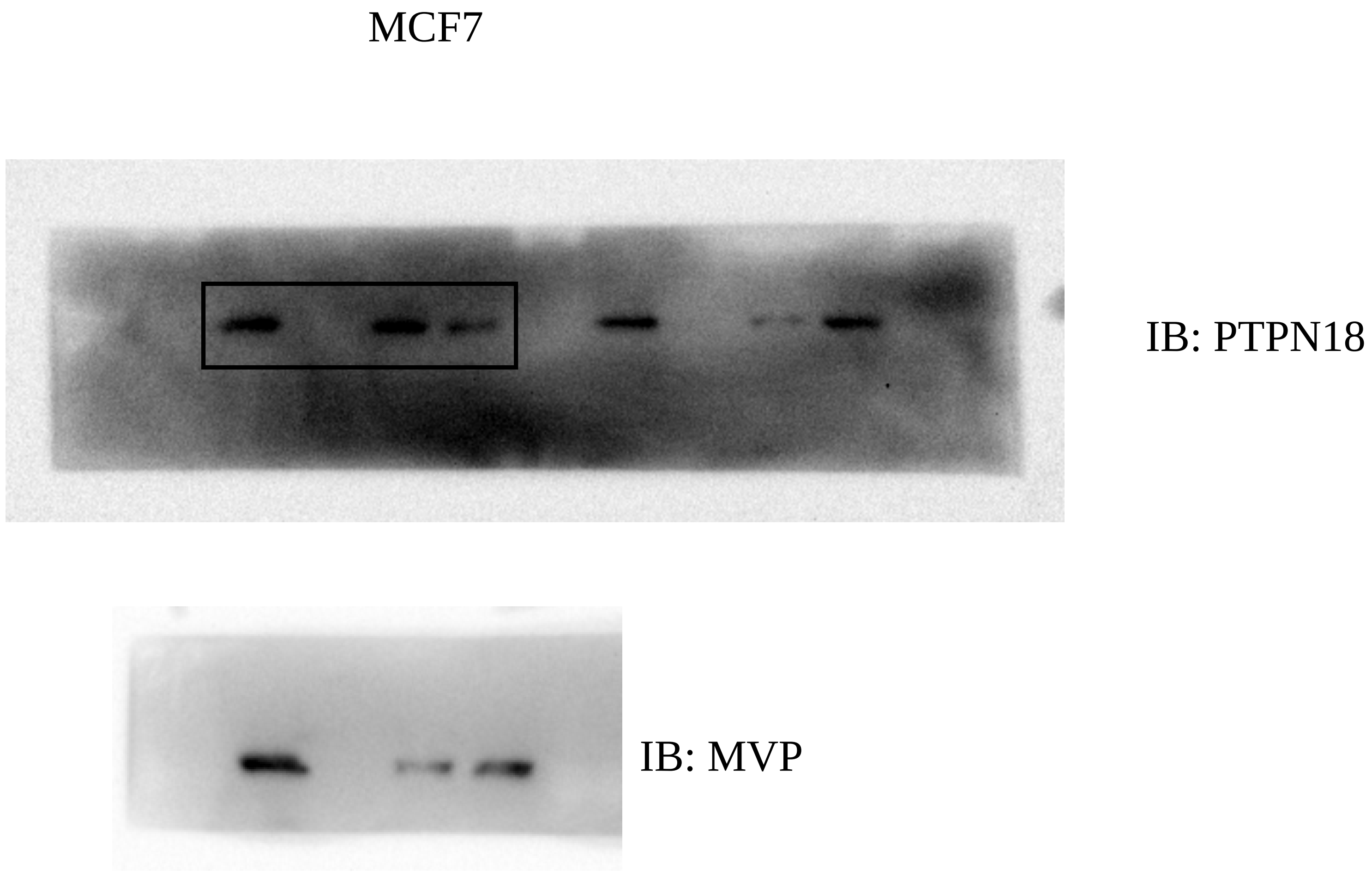

Fig. 1C

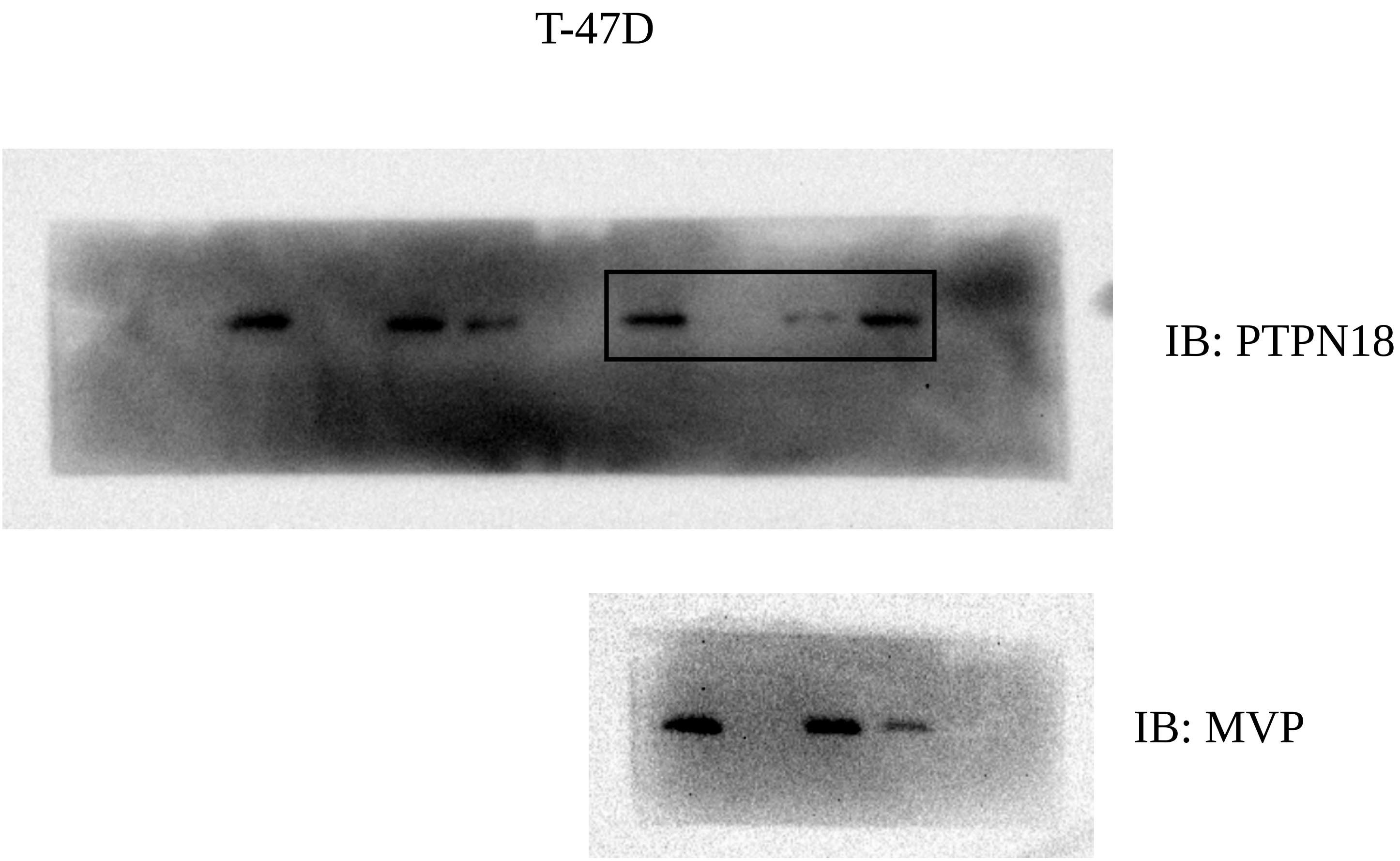

Fig. 1D

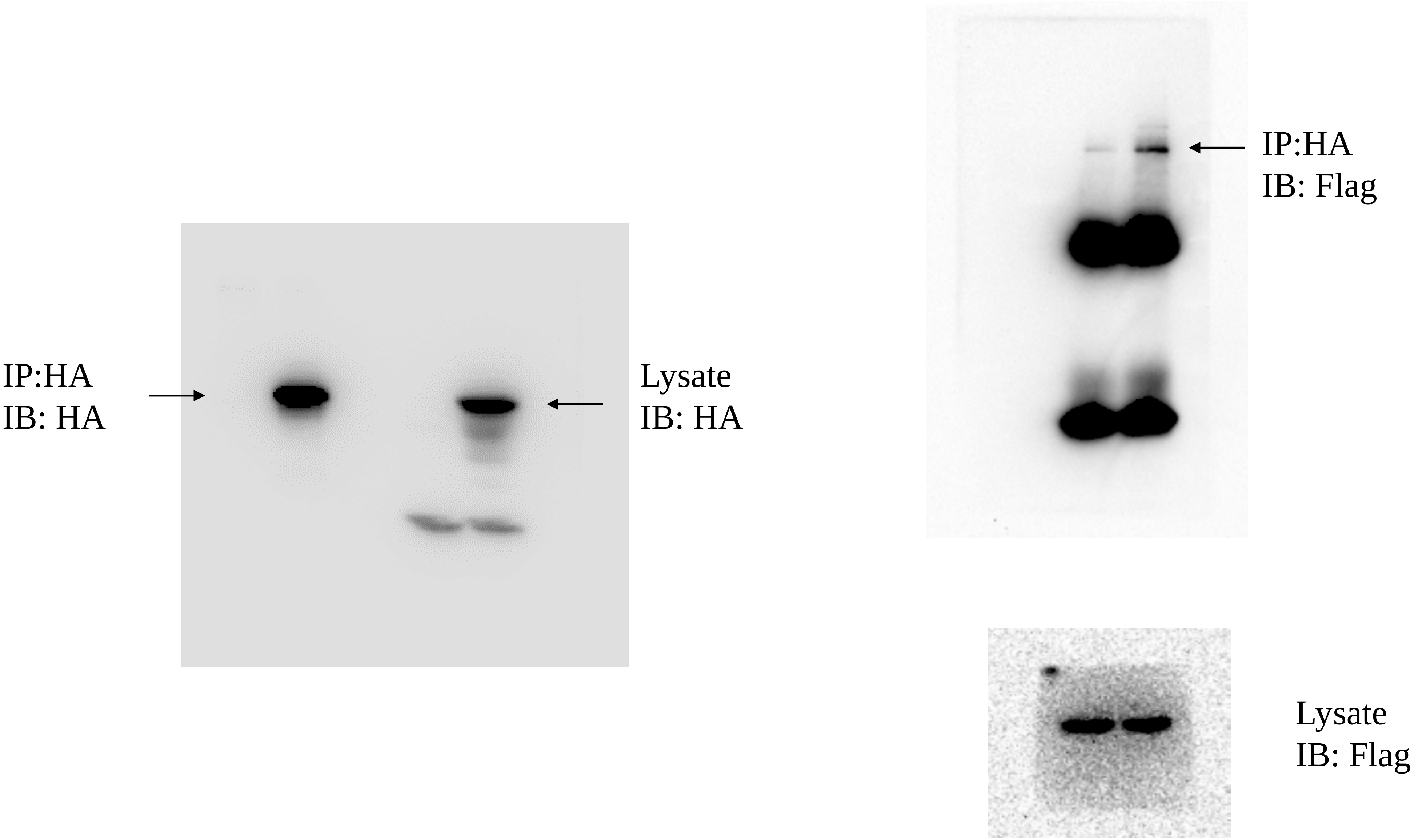

Fig. 1E

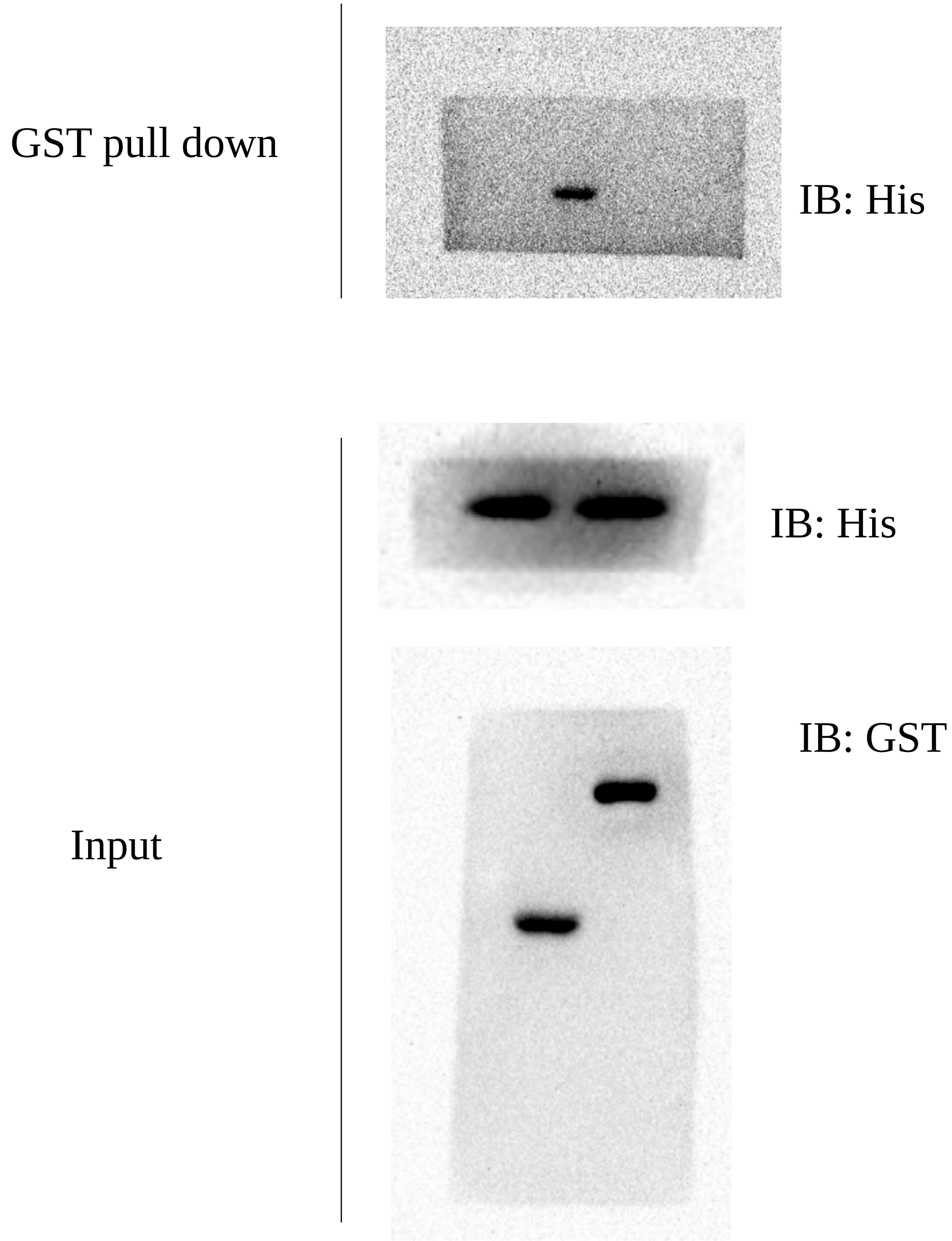

Fig. 2C

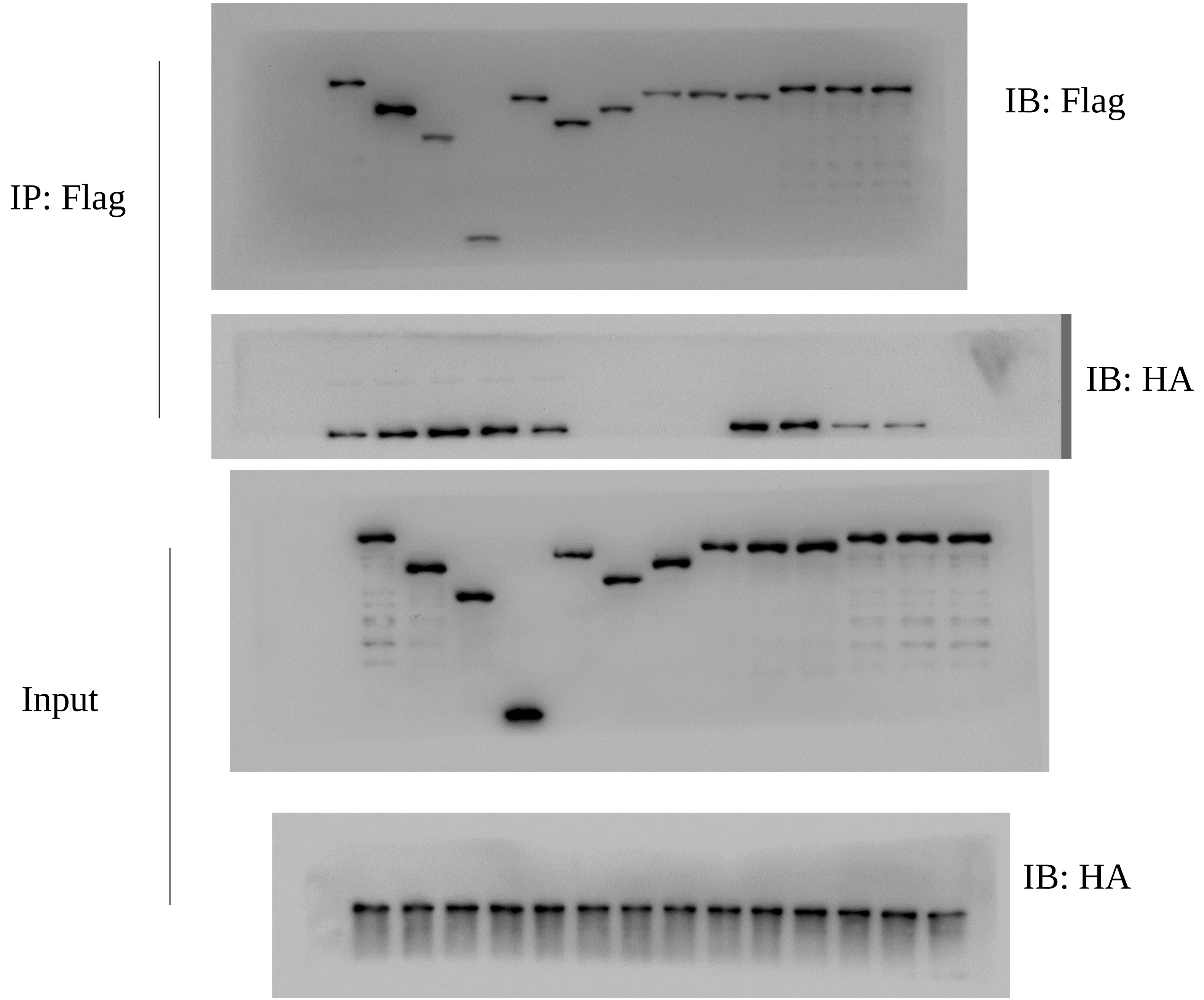

Fig. 2E

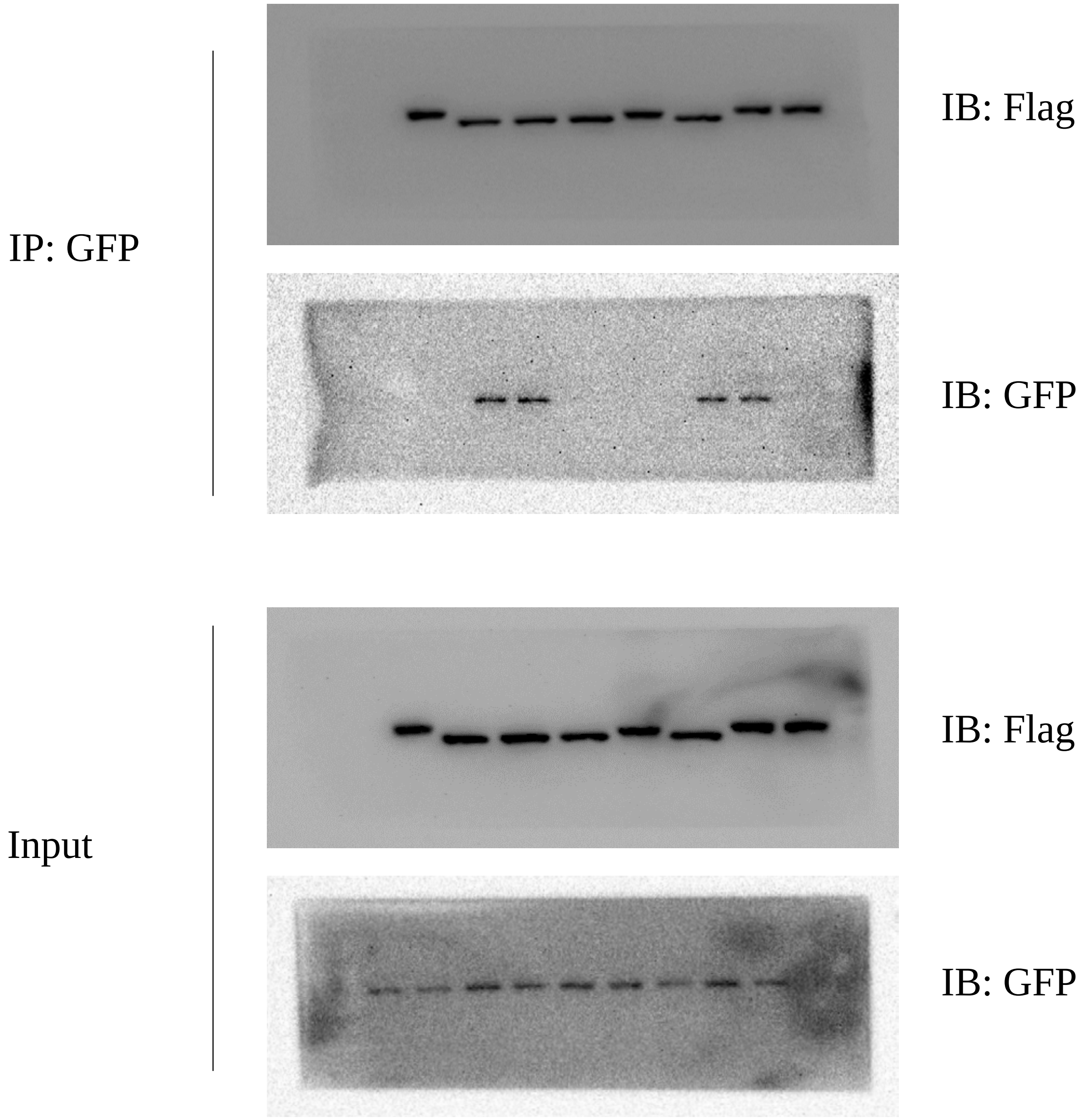

Fig. 3A

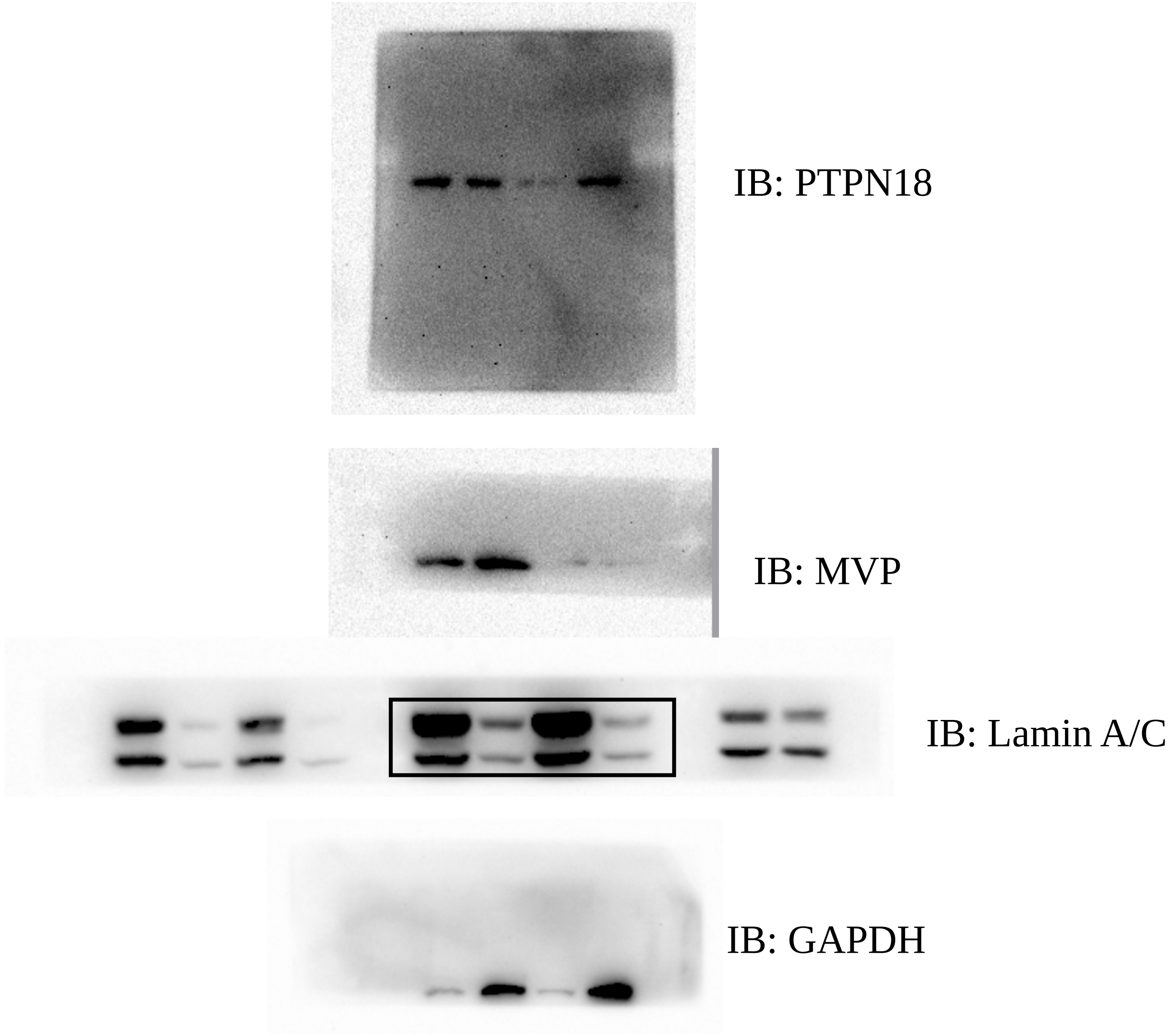

Fig. 3B

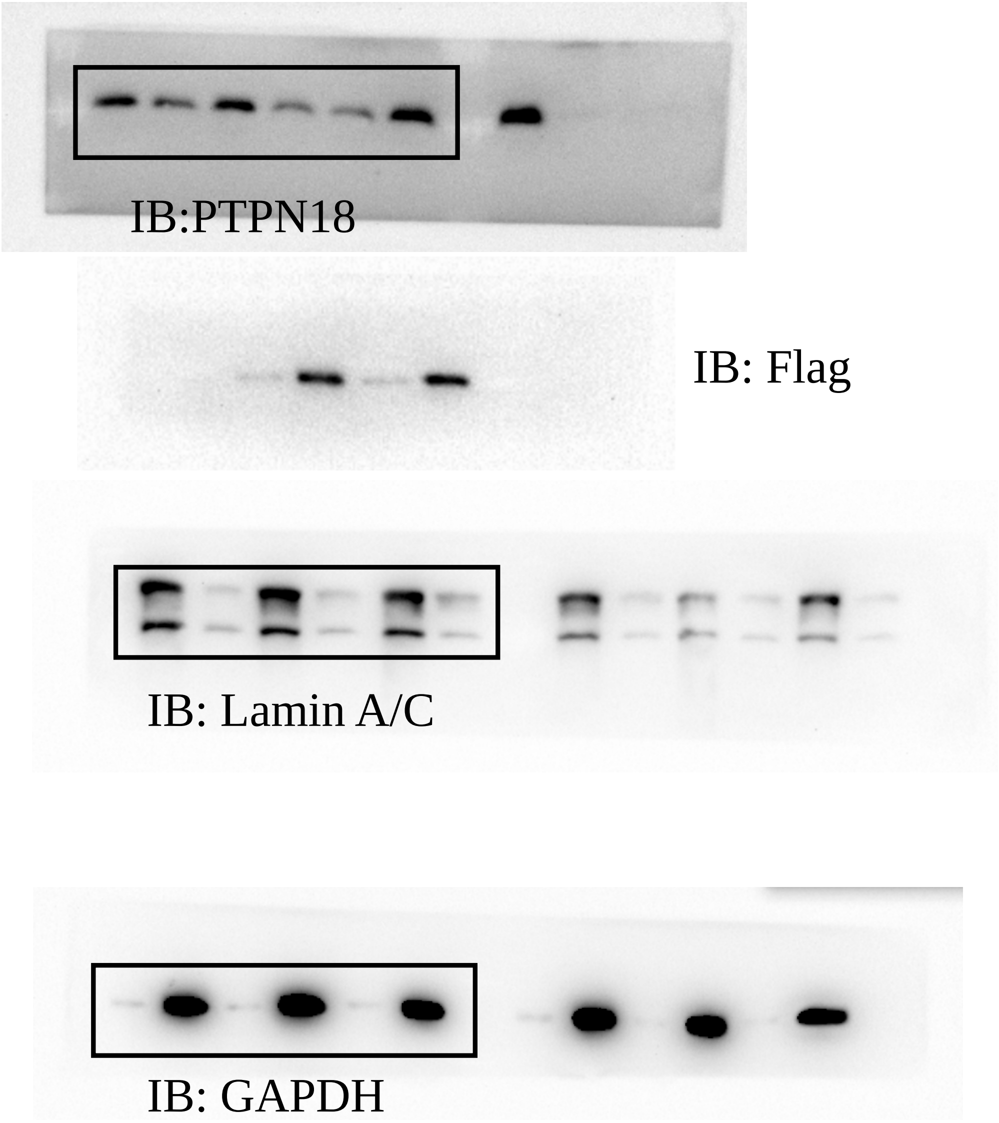

Fig. 3D

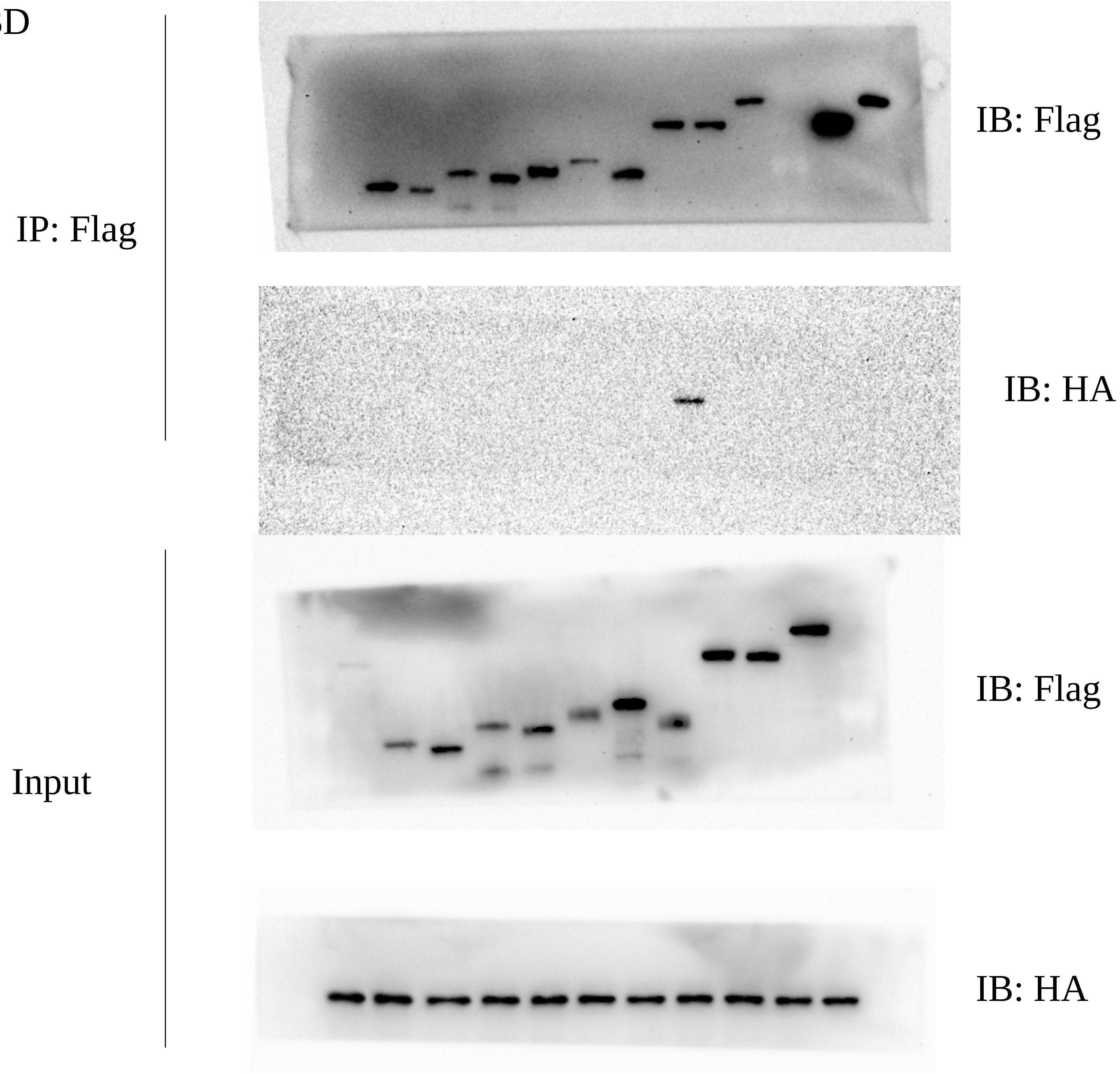

Fig. 3E

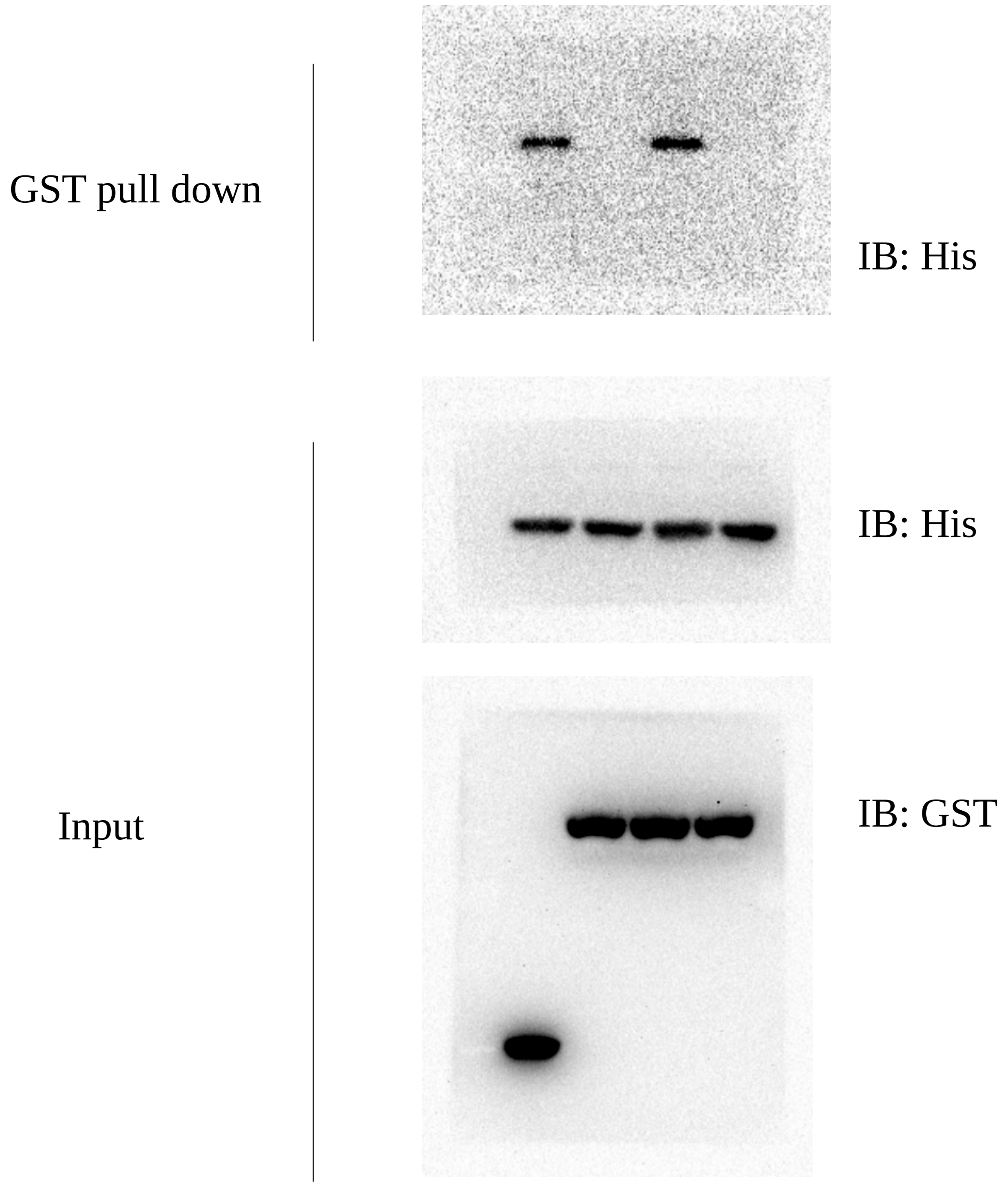

Fig. 4A

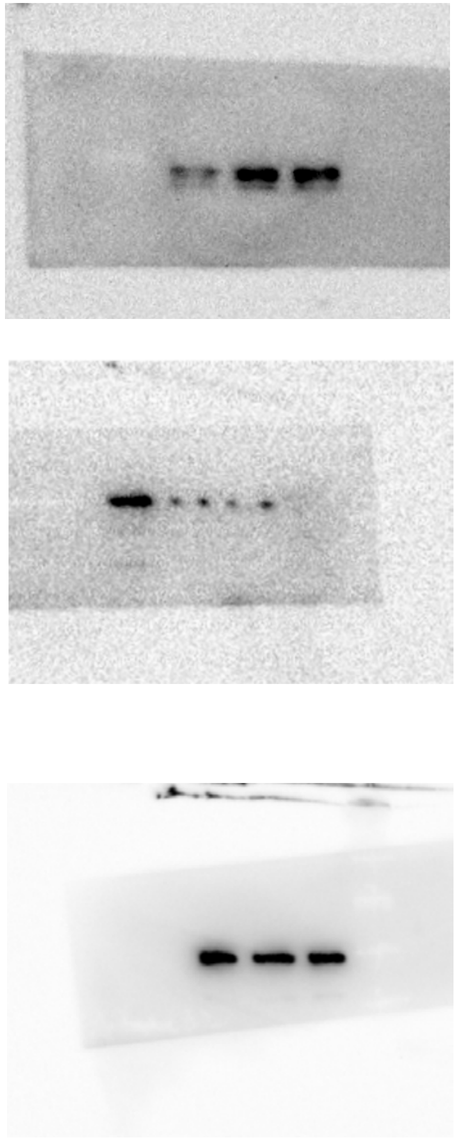

IB: ETS1

IB: PTPN18

IB: GAPDH

Fig. 4B

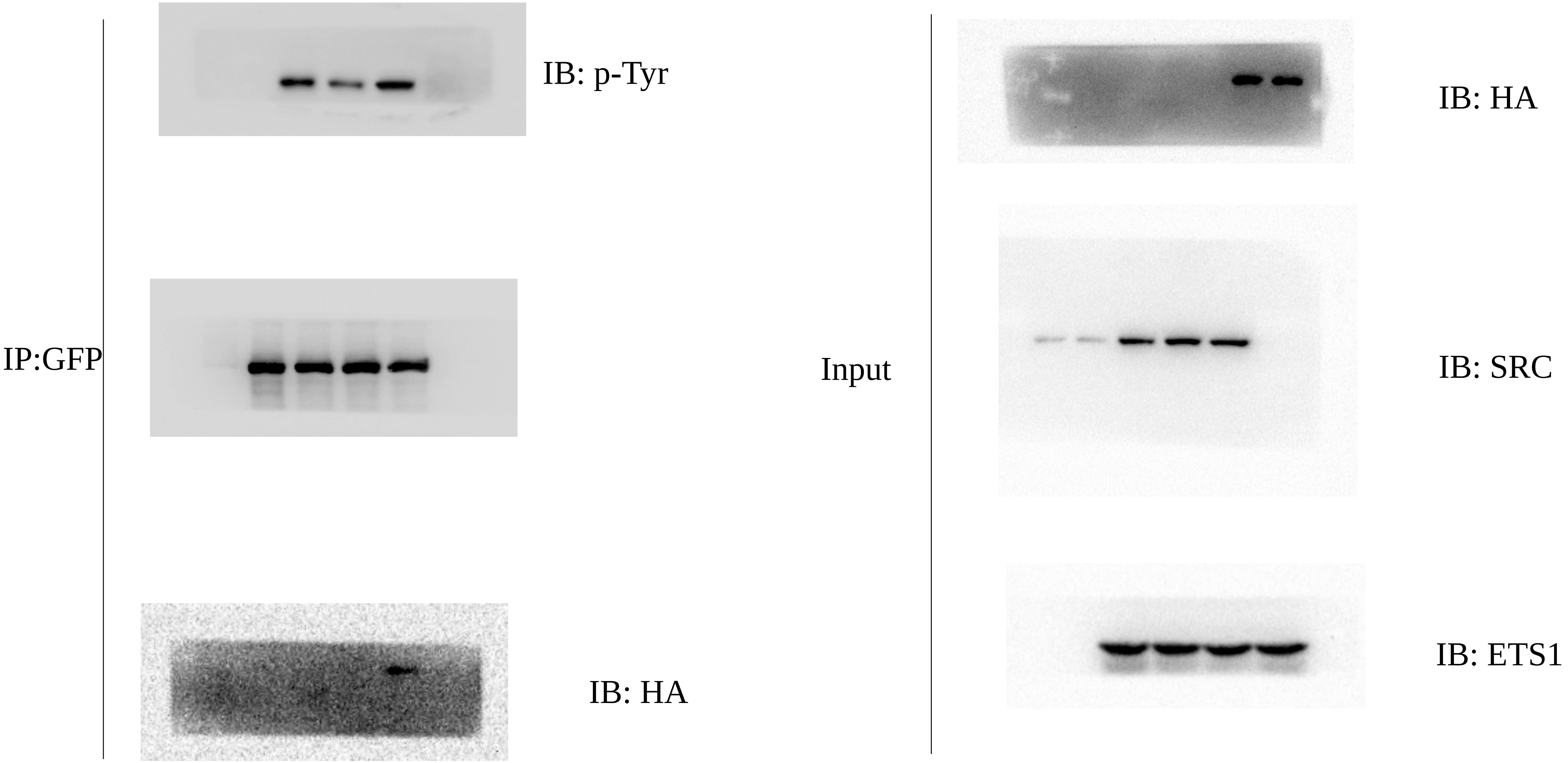

Fig. 4C

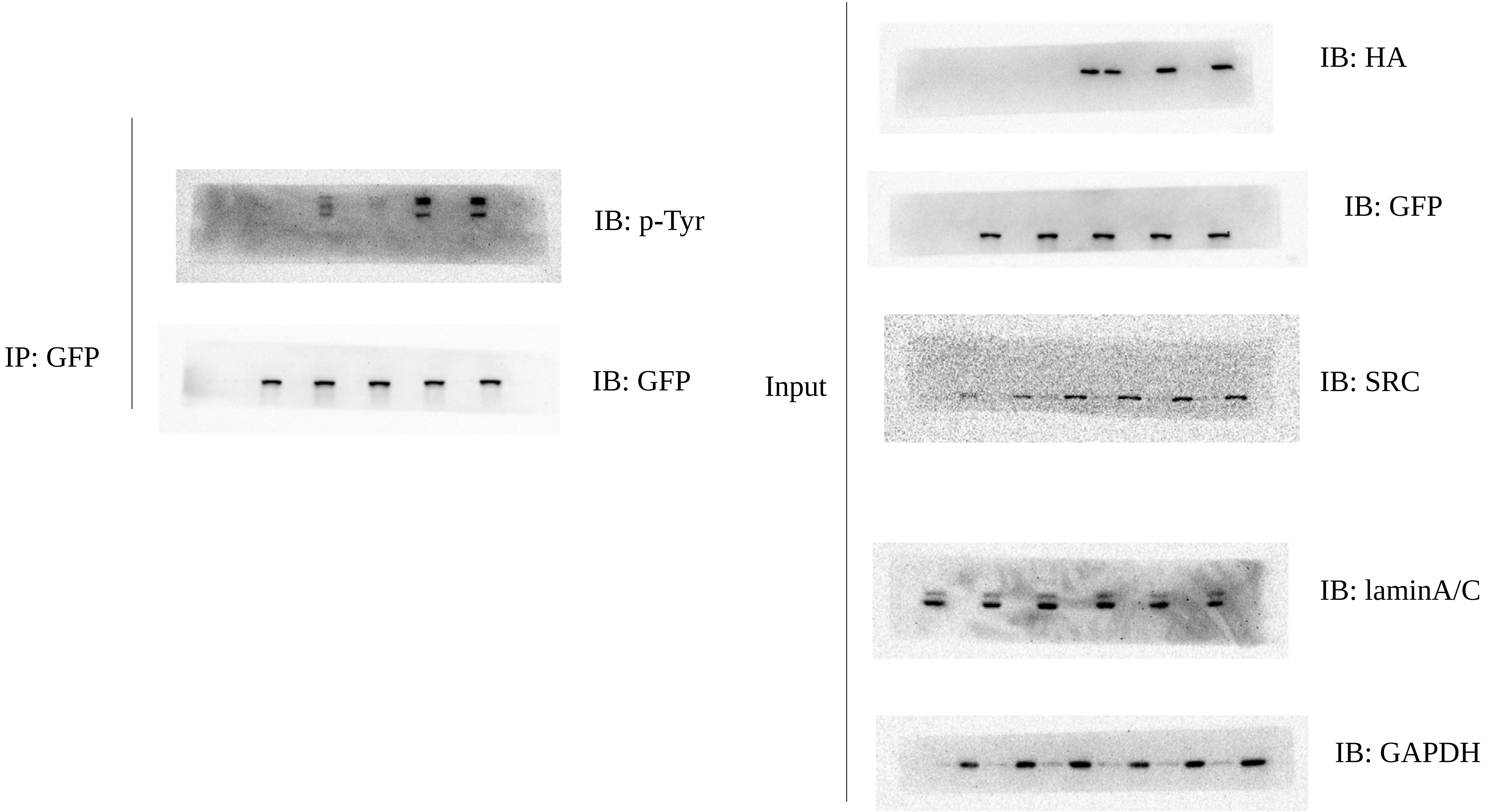
IB: p-Tyr
IB: GFP
IP: GFP
Input
IB: HA
IB: GFP
IB: SRC
IB: laminA/C
IB: GAPDH

Fig. 4D

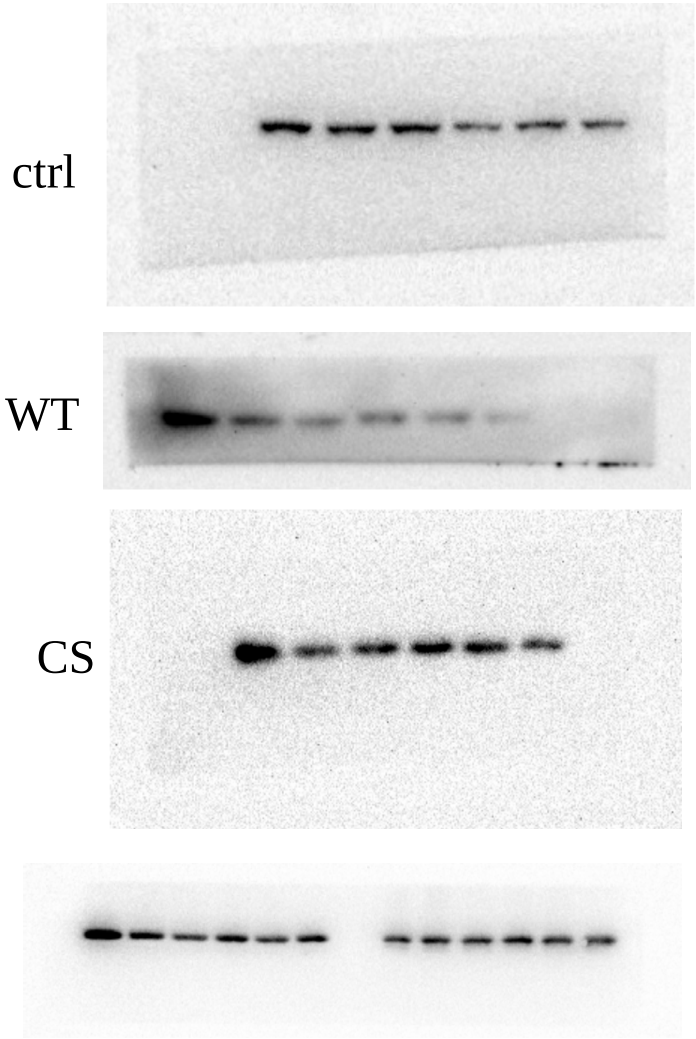

IB: ETS1

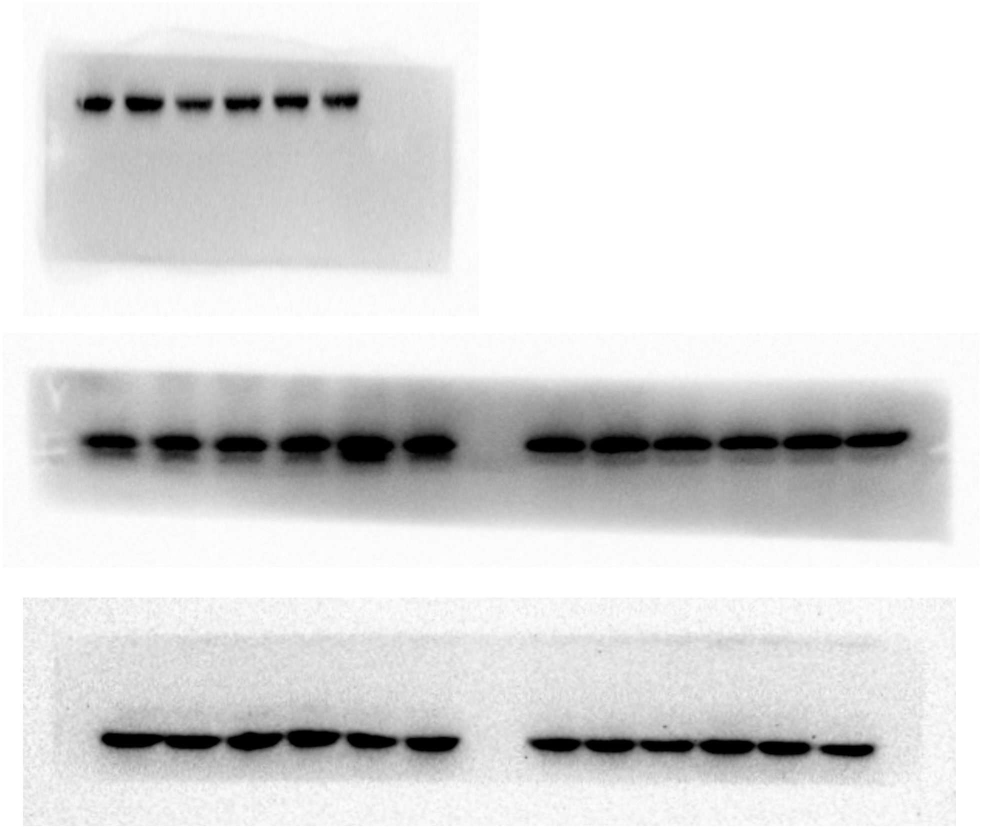

IB: GAPDH

Fig. 5A

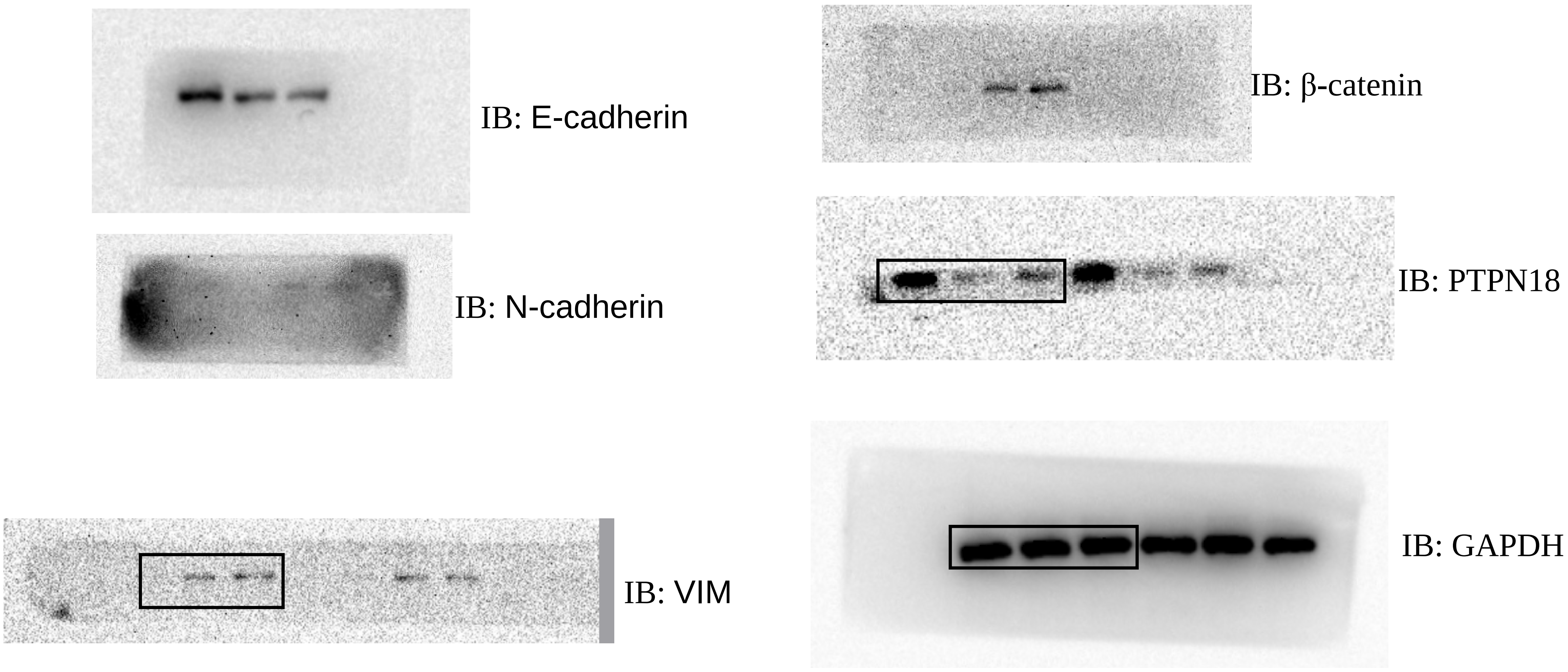
IB: E-cadherin
IB: N-cadherin
IB: VIM
IB: β-catenin
IB: PTPN18
IB: GAPDH

Fig. 5B

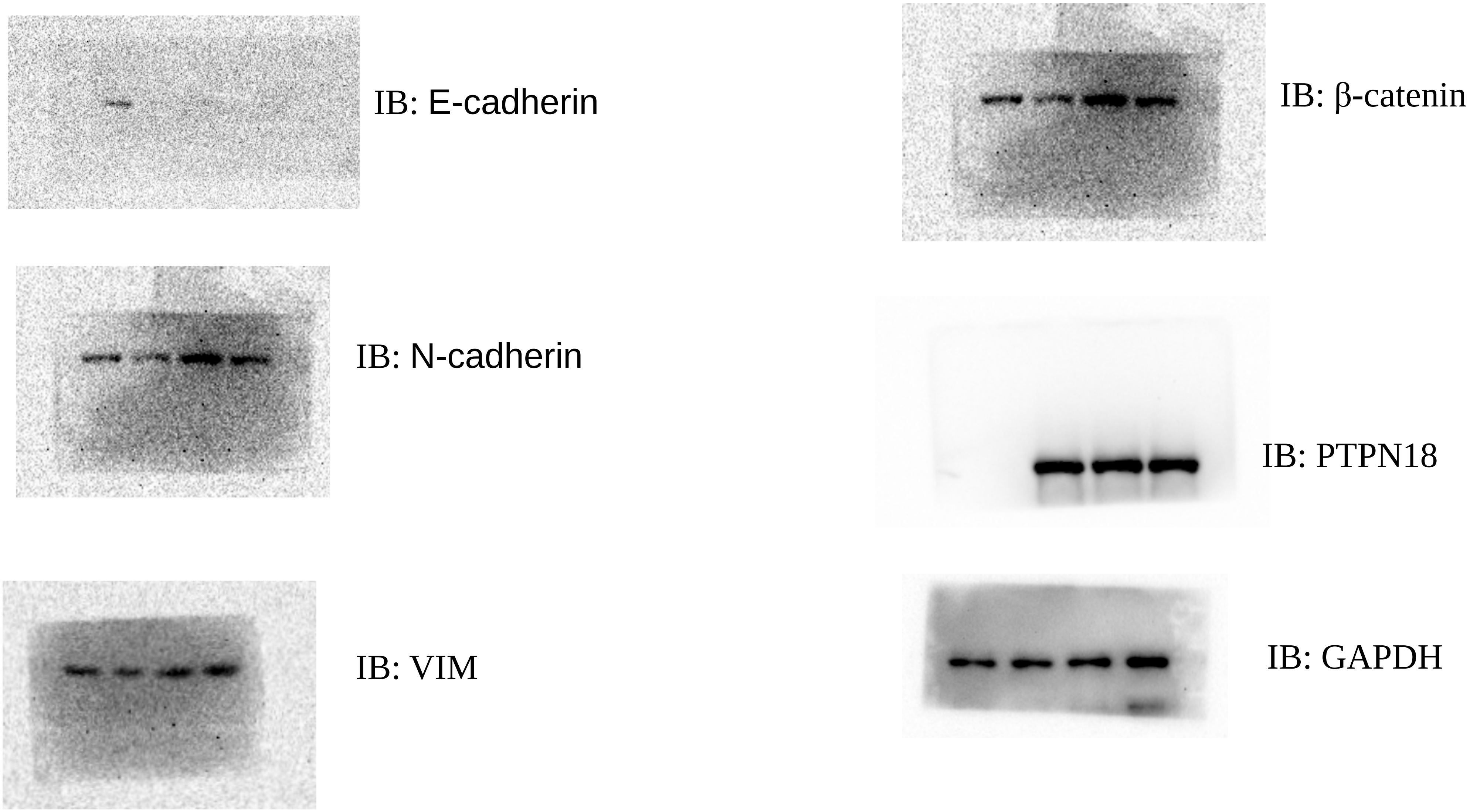
IB: E-cadherin
IB: β-catenin
IB: N-cadherin
IB: PTPN18
IB: VIM
IB: GAPDH

Fig. 5D

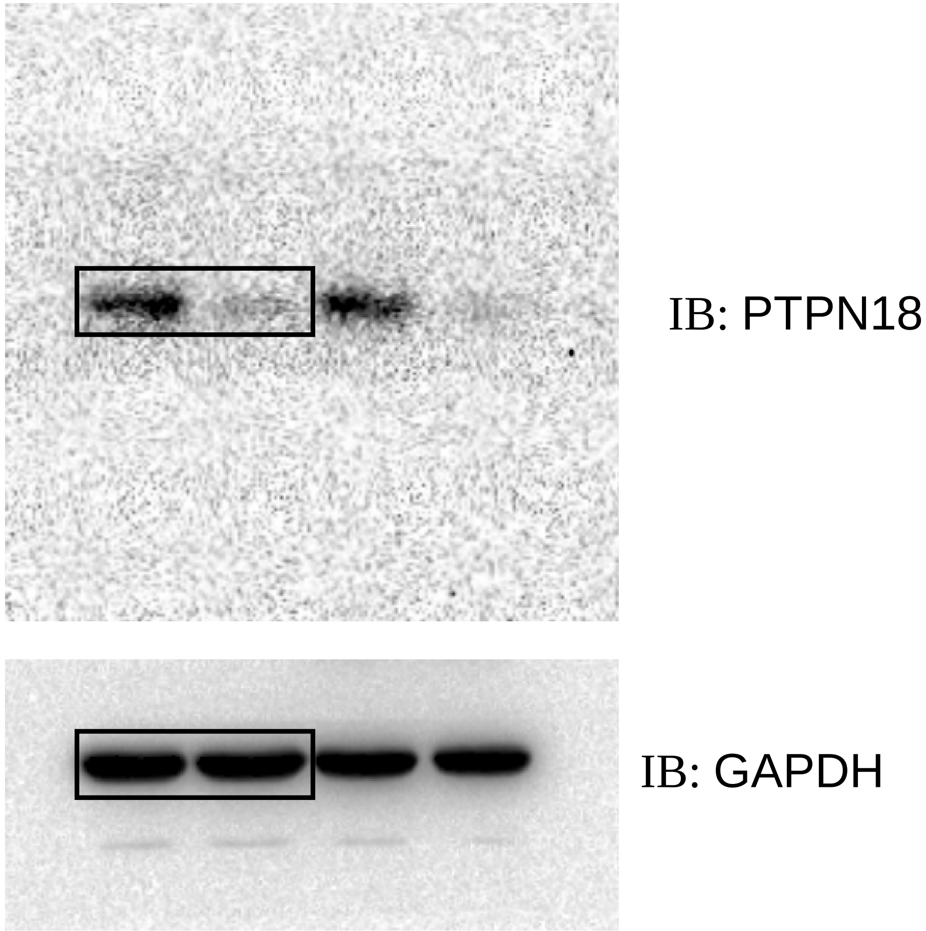

Fig. 5F

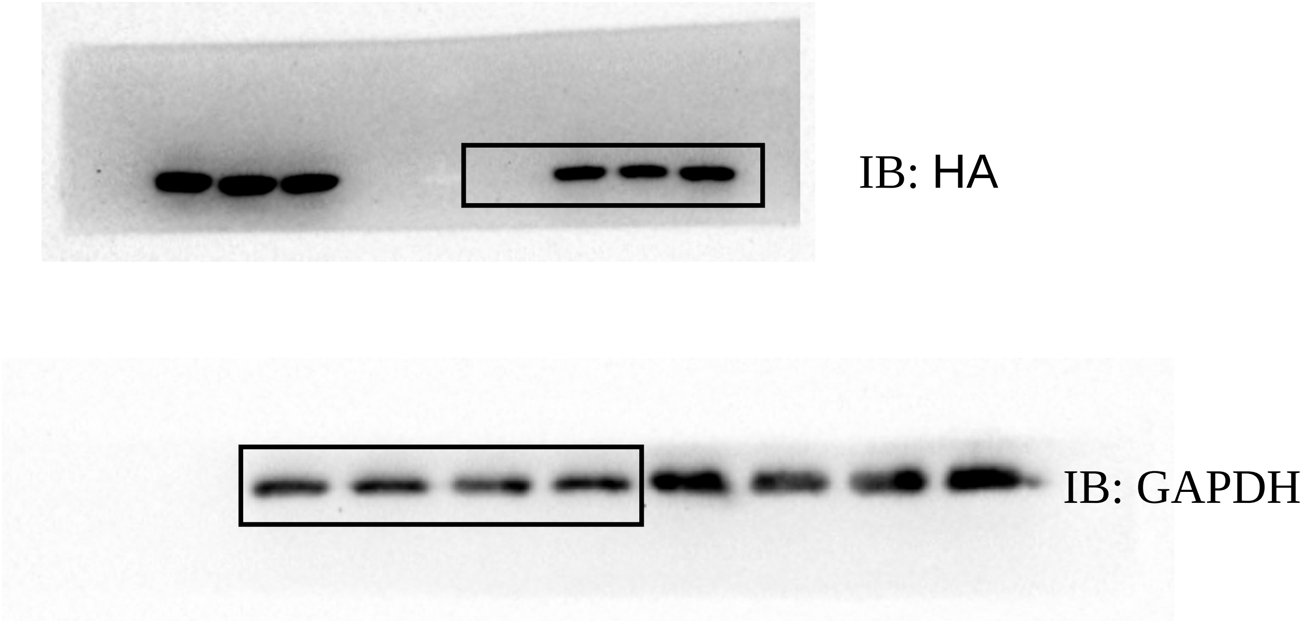

Fig. S1D

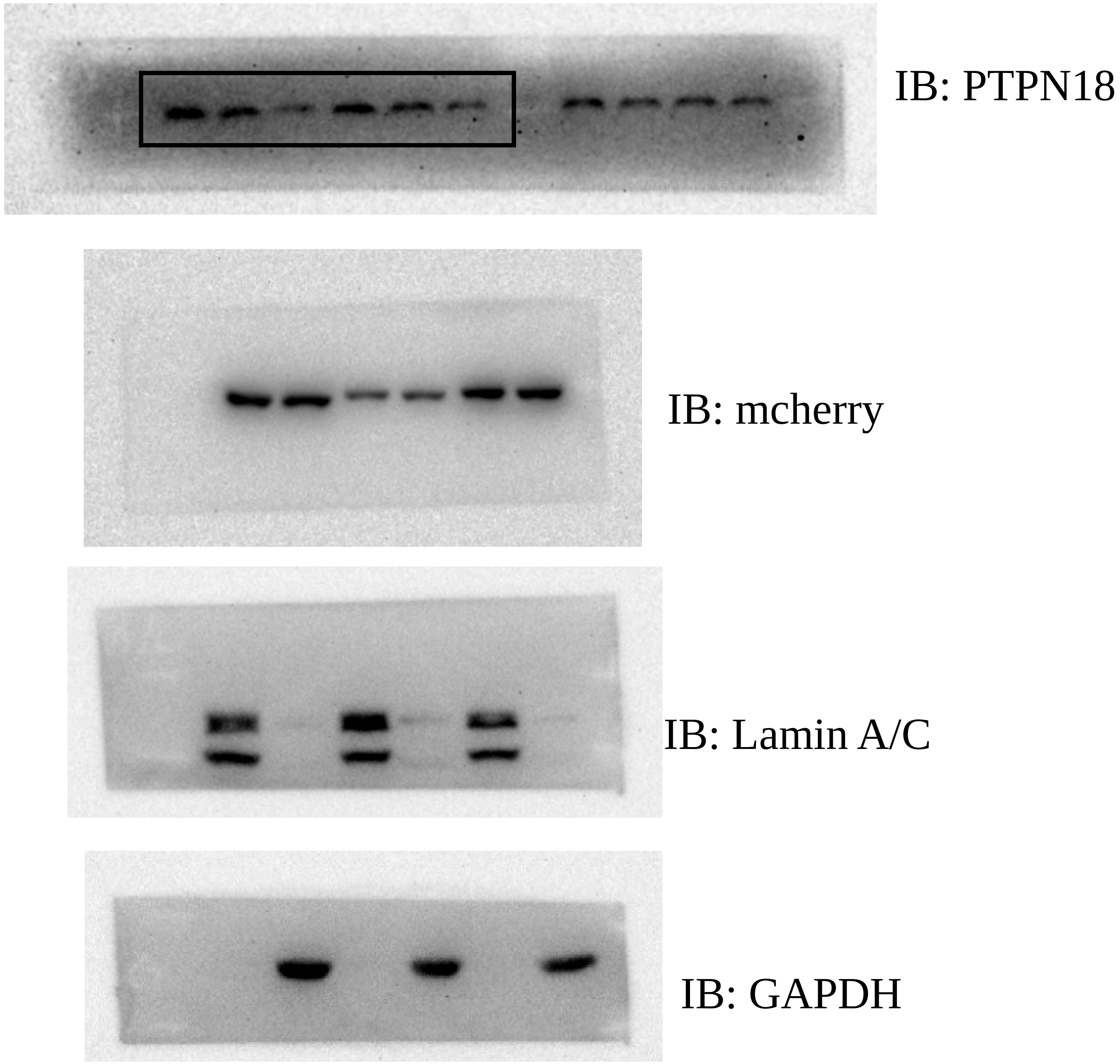

Fig. S1F

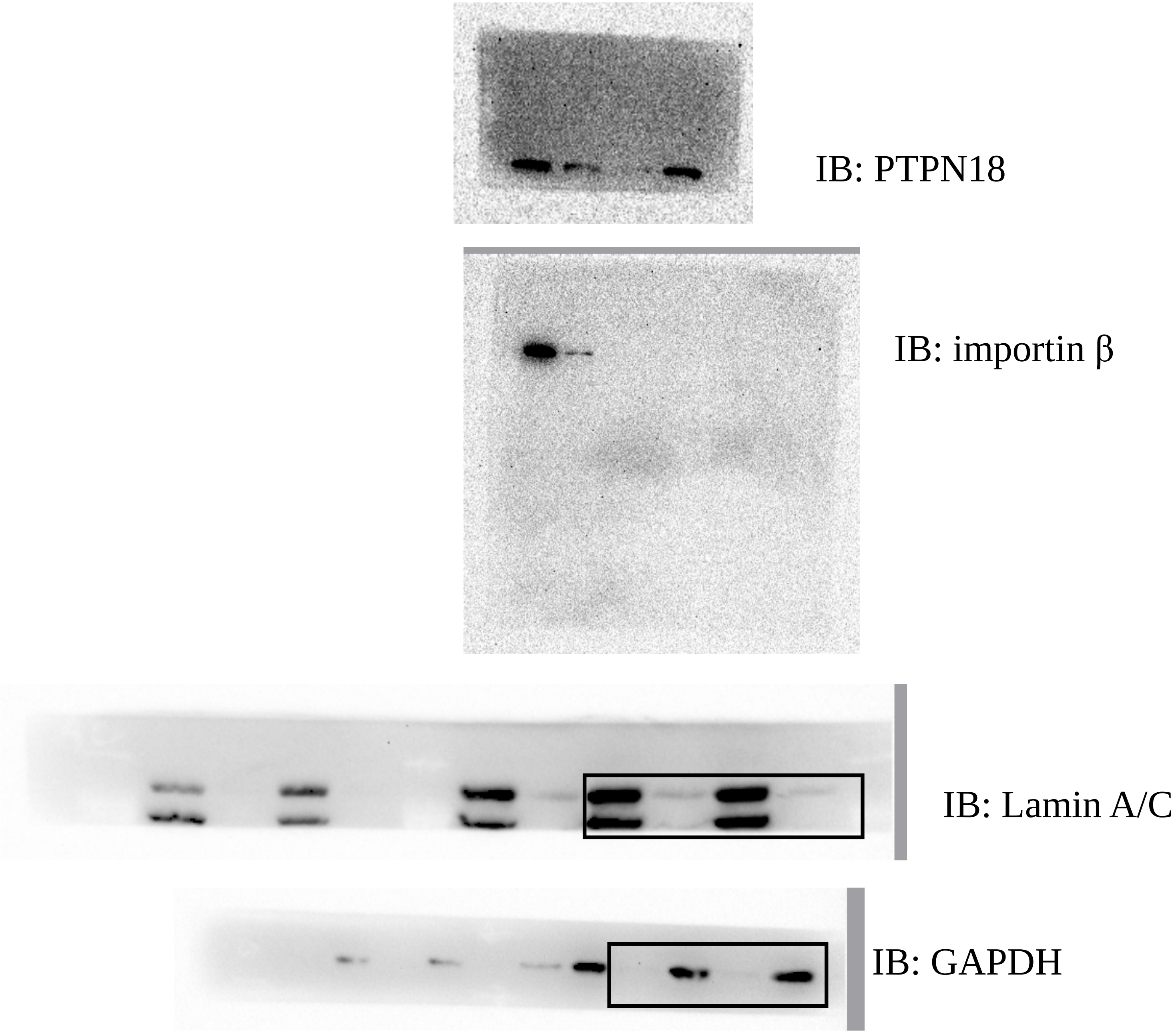

Fig. S3C

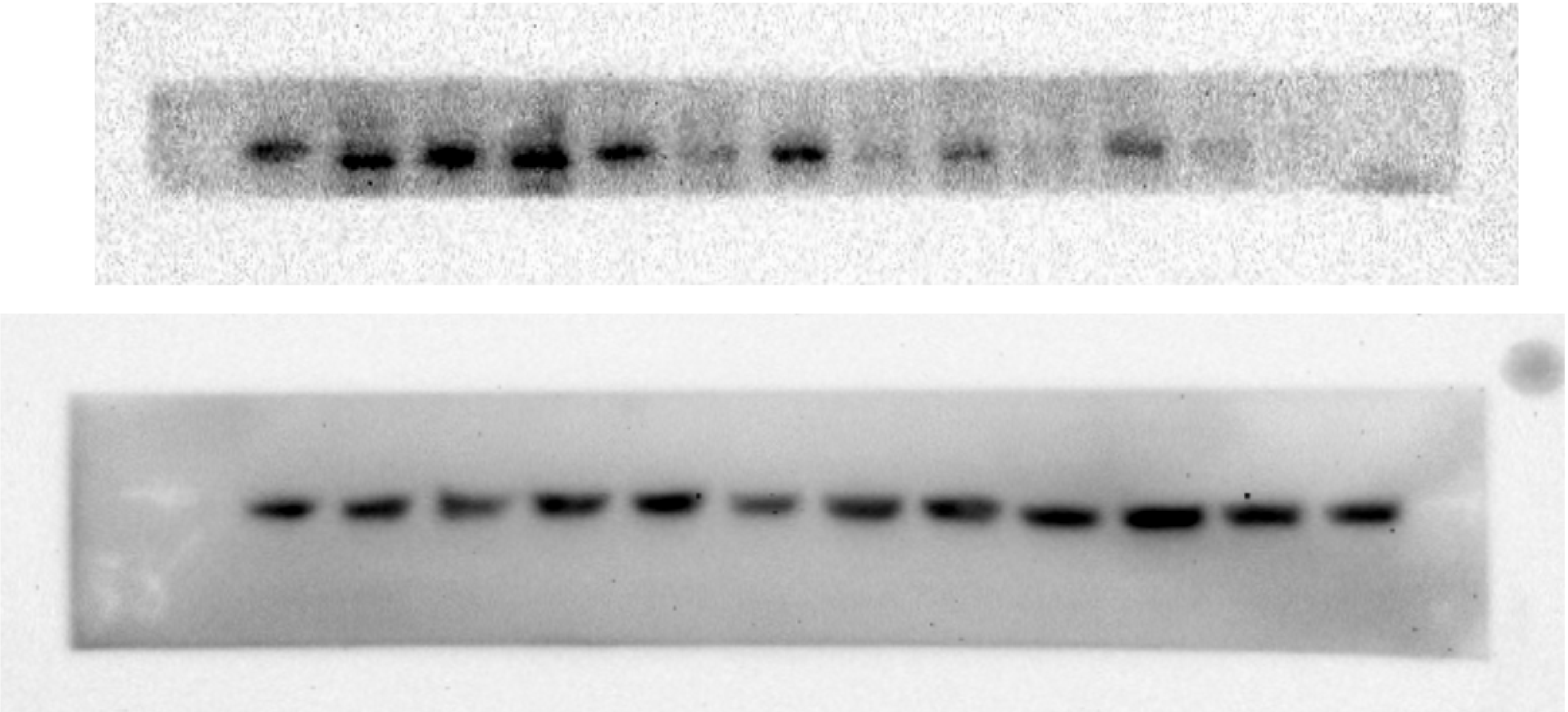

IB: N18

IB: GAPDH

Fig. S3G

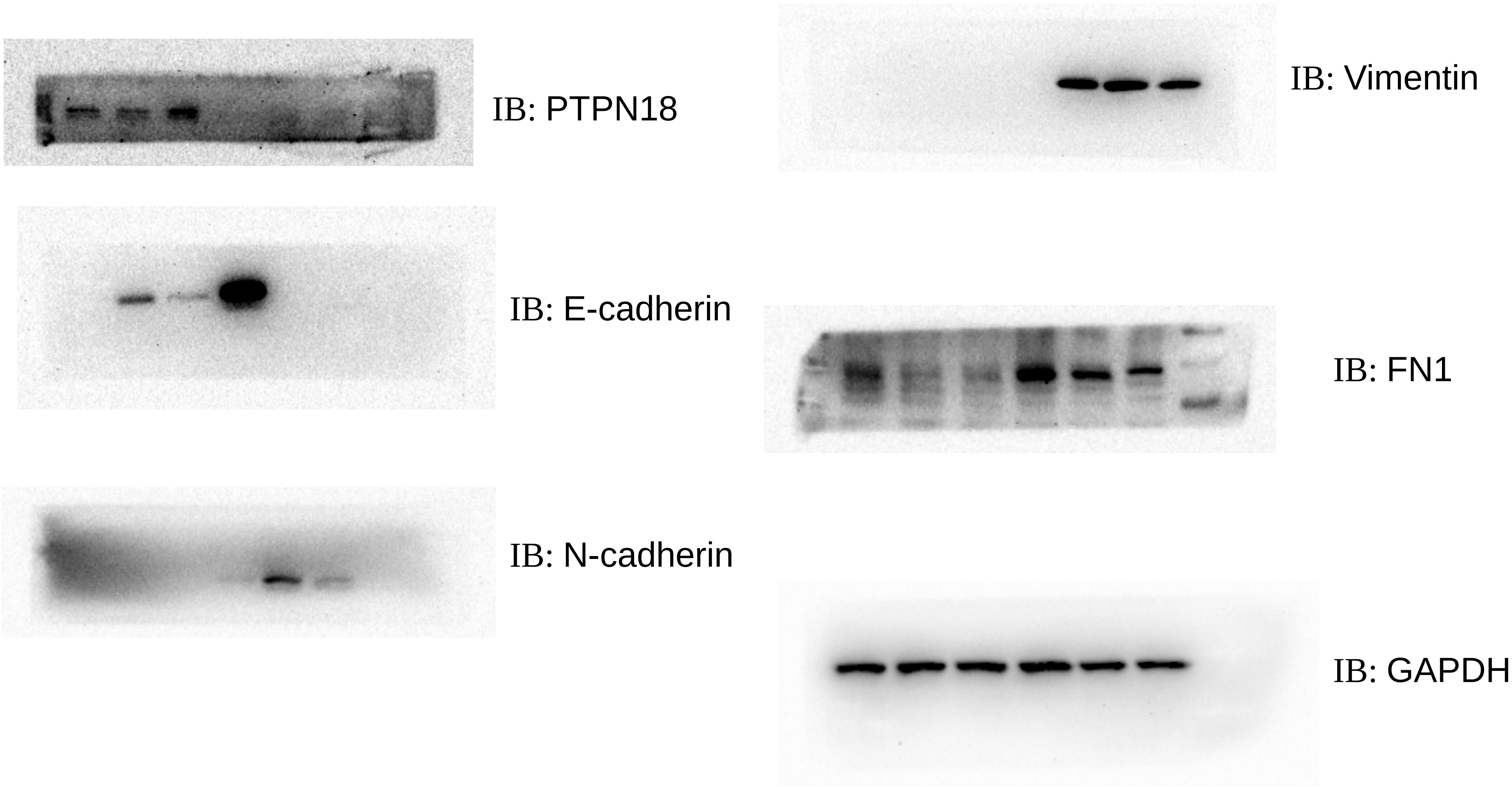
IB: PTPN18
IB: E-cadherin
IB: N-cadherin
IB: Vimentin
IB: FN1
IB: GAPDH

Fig. 5A

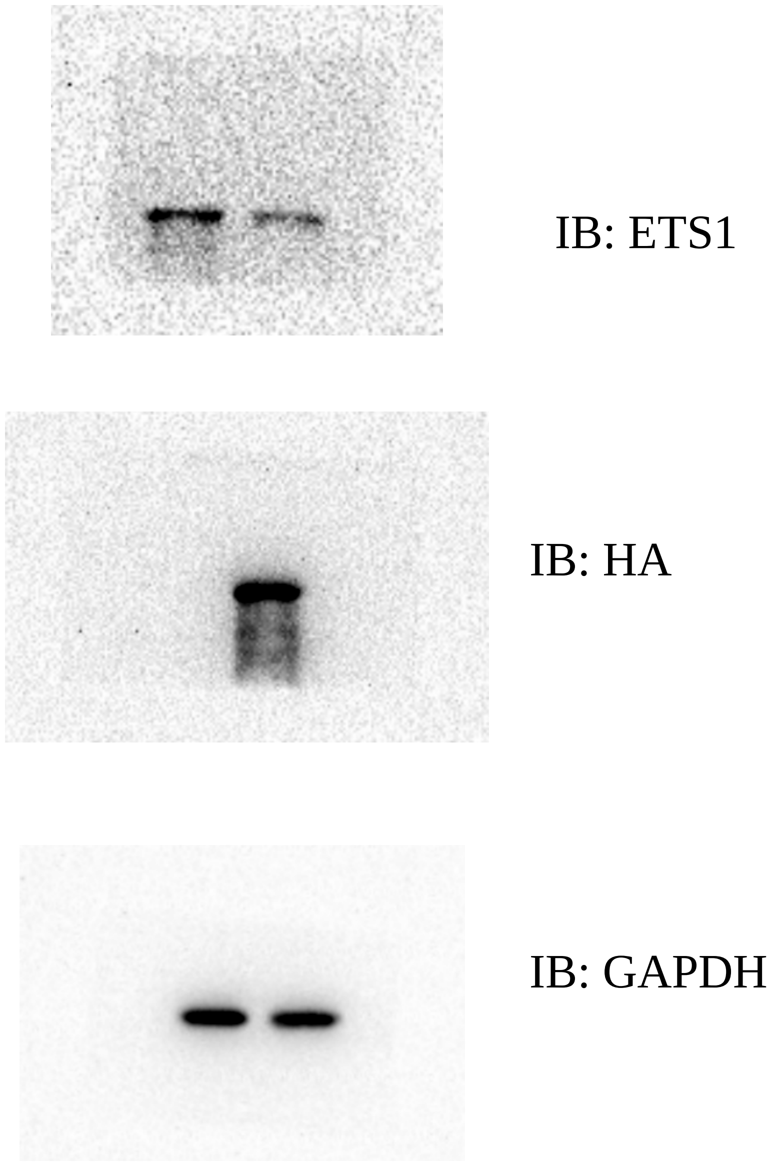

Fig. 5B

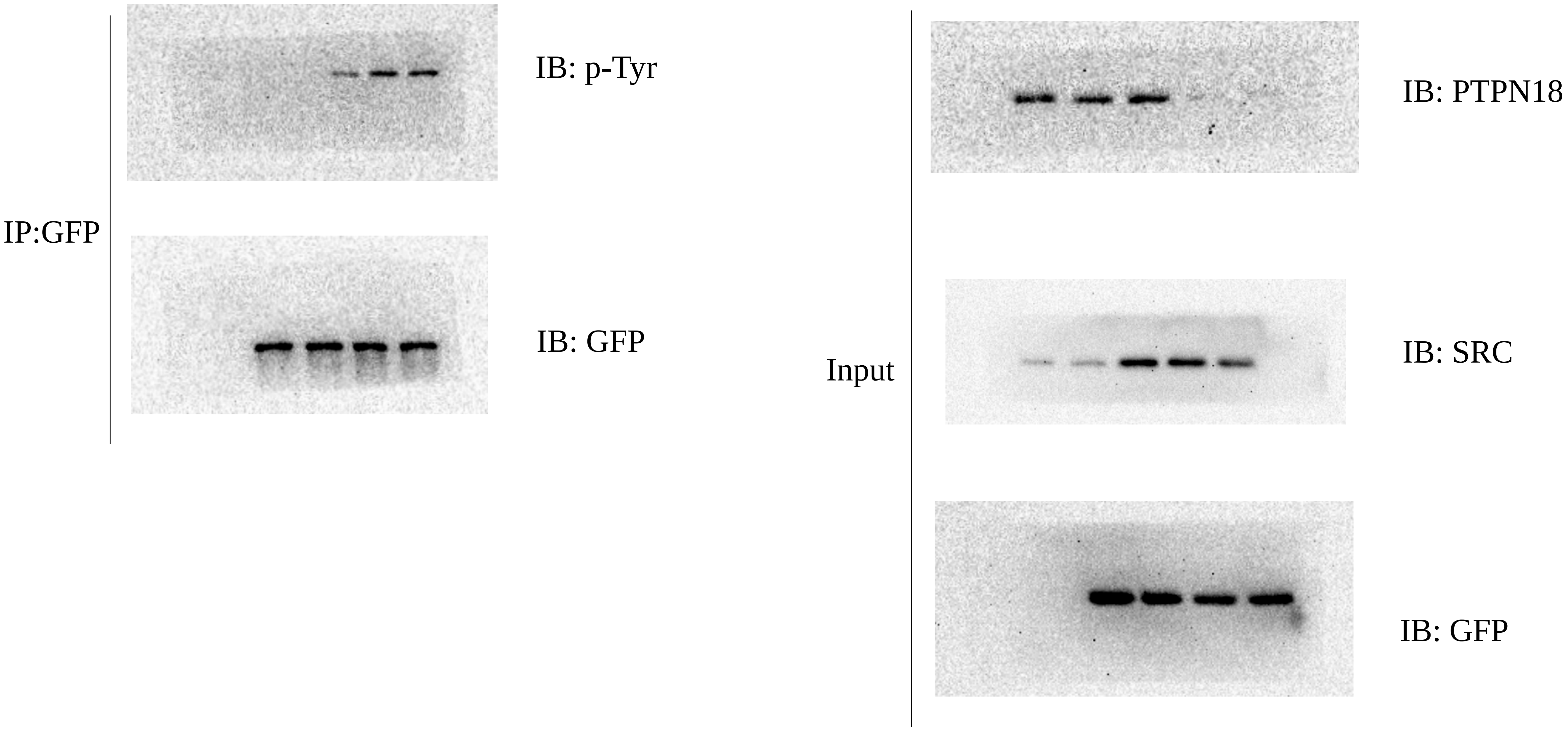

Fig. S6A

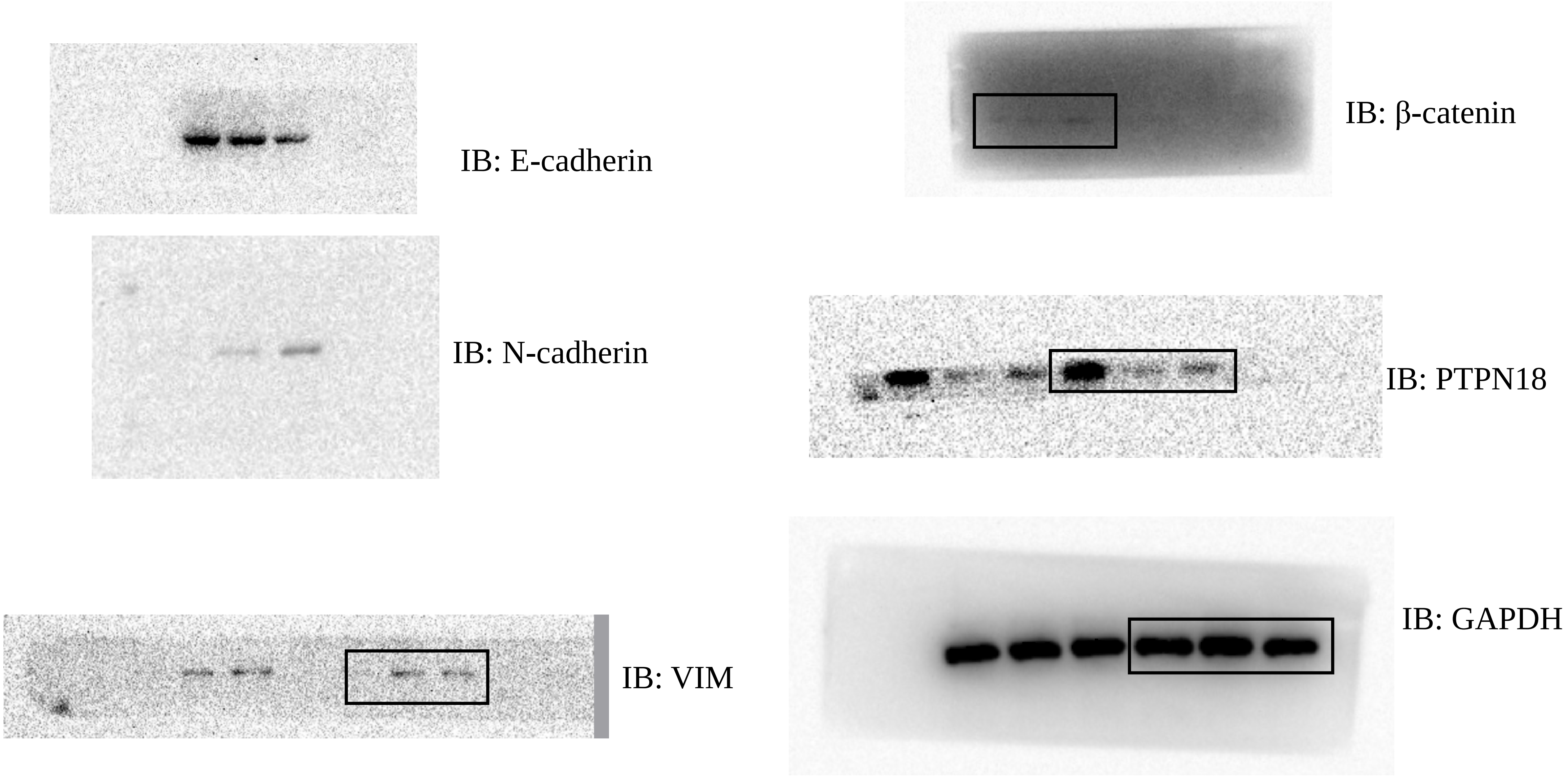
IB: E-cadherin
IB: N-cadherin
IB: VIM
IB: β-catenin
IB: PTPN18
IB: GAPDH

Fig. S6B

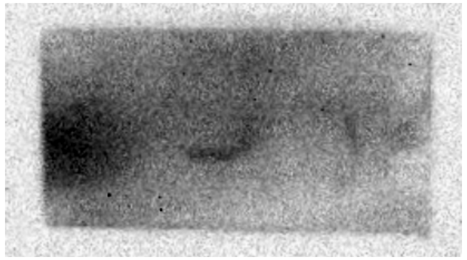

IB: E-cadherin

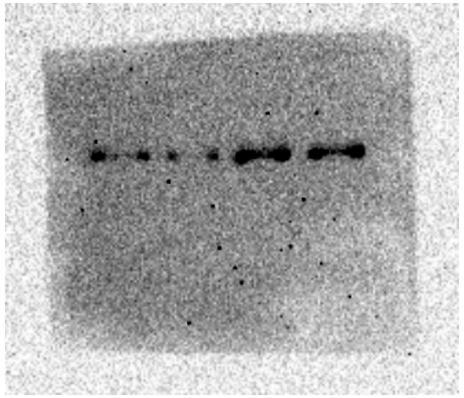

IB: N-cadherin

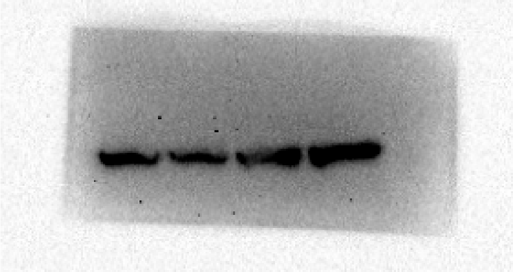

IB: Vimentin

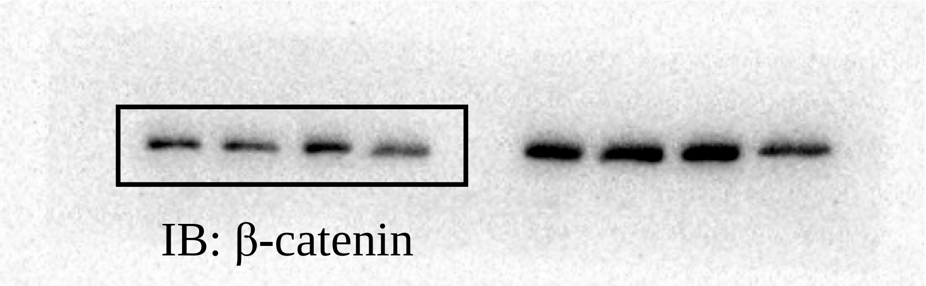

IB: β-catenin

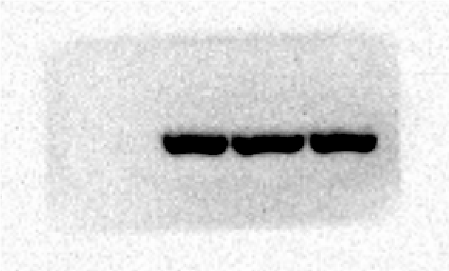

IB: PTPN18

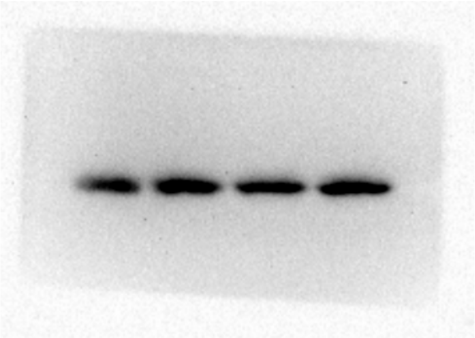

IB: GAPDH

Fig. S6D

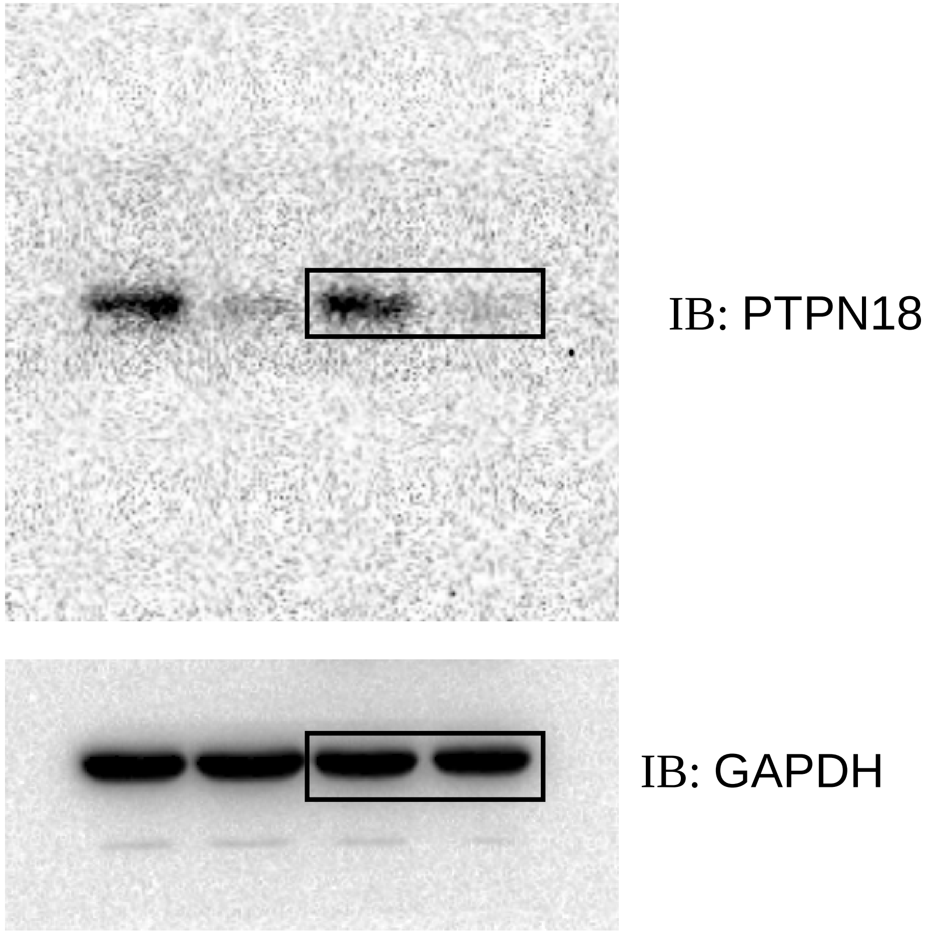

Fig. S6F

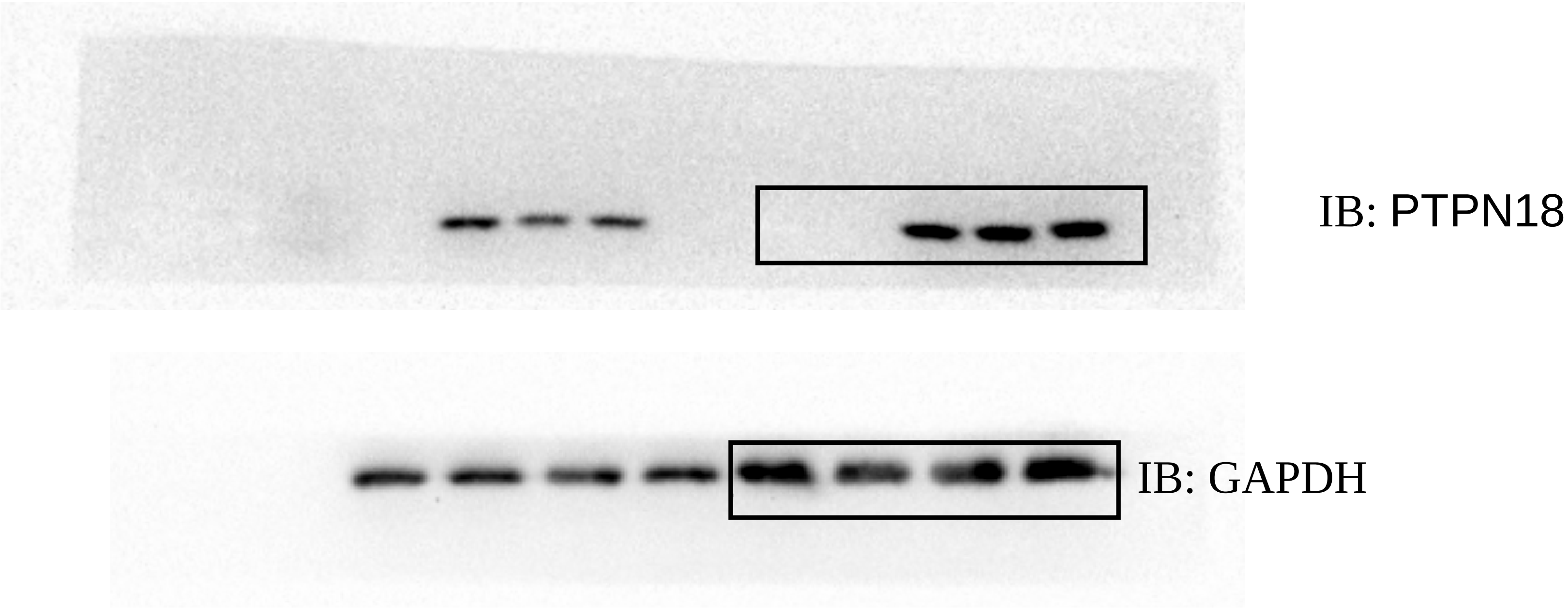

Fig. S7A

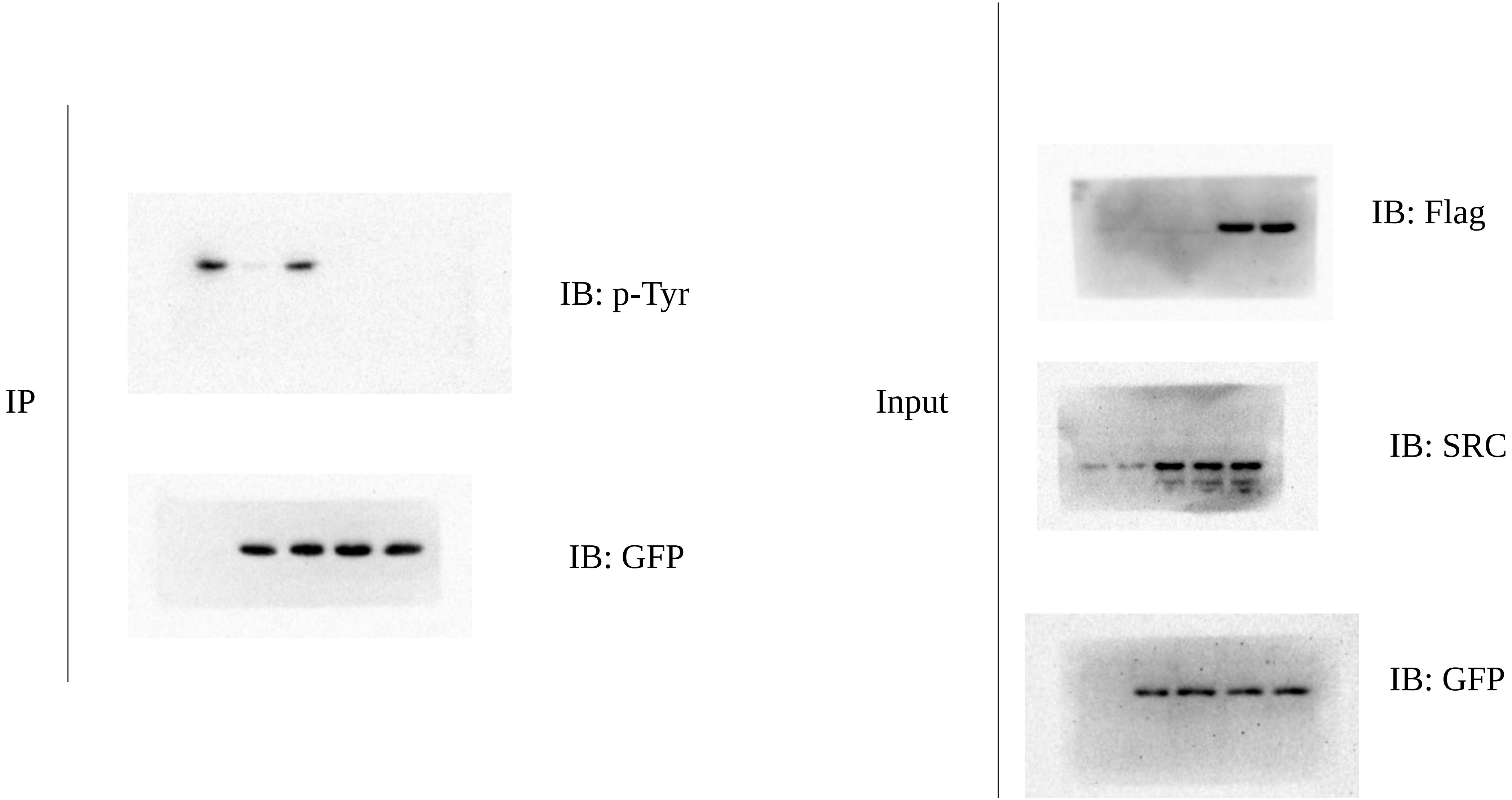

Fig. S7B

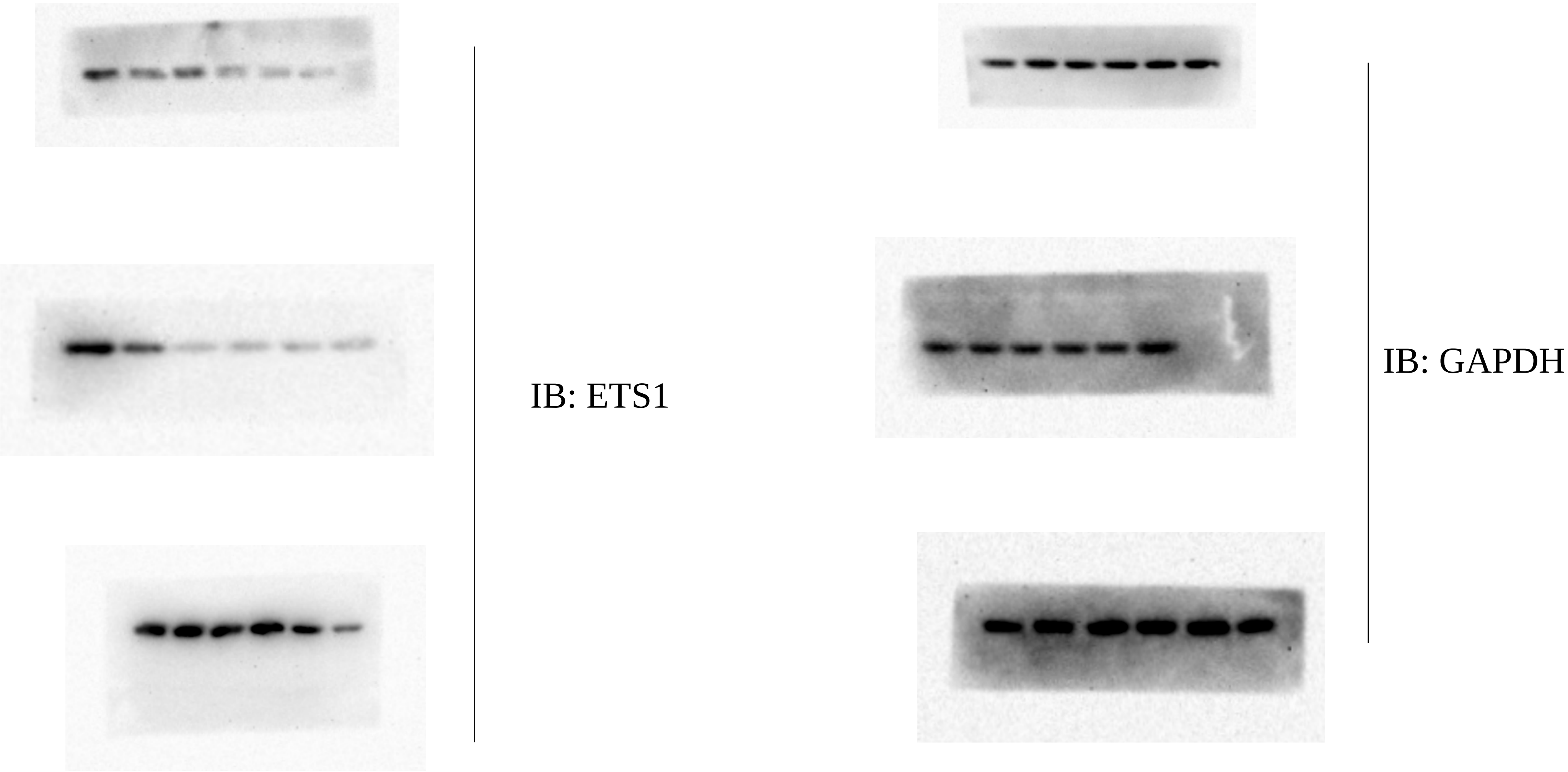

Fig. S7C

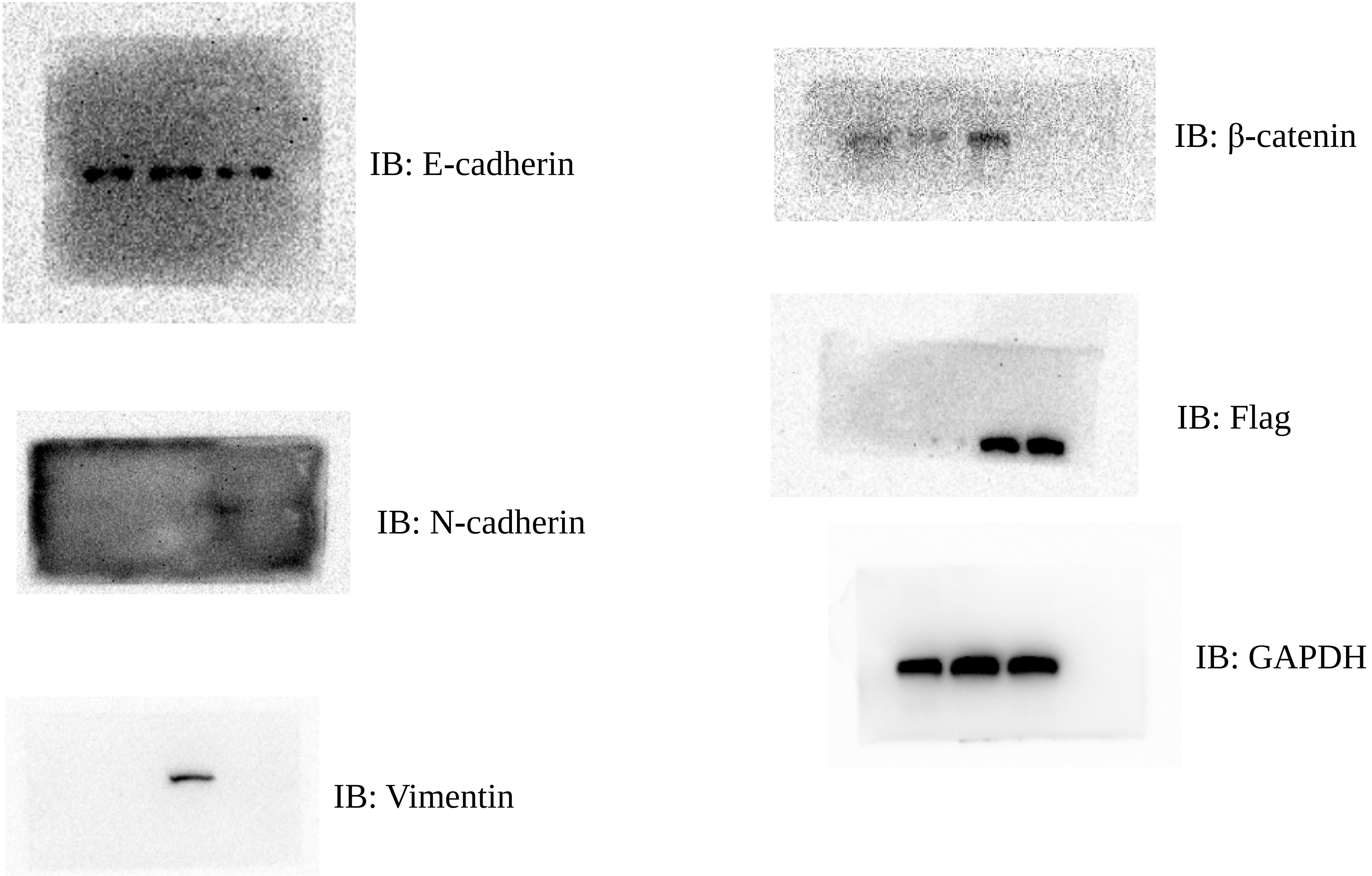
IB: E-cadherin
IB: β-catenin
IB: Flag
IB: N-cadherin
IB: GAPDH
IB: Vimentin
